# Electrochemically Driven Microbial Anode-Membrane Capacitor Deionization System: Energy Consumption Analysis for Enhancing NaCl Removal and Desalination at Different Gradients

**DOI:** 10.3390/membranes16080263

**Published:** 2026-08-07

**Authors:** Wenlong Liu, Jun Pan

**Affiliations:** School of Municipal and Environmental Engineering, Shenyang Jianzhu University, Shenyang 110168, China

**Keywords:** microbial anodic-membrane capacitive deionization (B-MCDI), microbial electrochemical desalination, Shewanella oneidensis MR-1, initial salt concentration, instantaneous desalination rate

## Abstract

To overcome the limitations of insufficient driving force in traditional microbial desalination batteries, this paper constructs a microbial anode-membrane capacitive deionization (B-MCDI) coupling system. For the first time, direct coupling between extracellular electron transfer in Shewanella oneidensis and double-layer adsorption at the MCDI cathode is achieved at the circuit and material levels, realizing self-driven, low-energy desalination. High-specific-surface-area carbon felt is used as the anode, and a stable electrogenic biomembrane (output voltage >400 mV) is formed after directional domestication with Shewanella oneidensis MR-1. Activated carbon is used as the cathode to construct the MCDI electrode. In the three-chamber reactor, the desalination chambers are separated by cation and anion exchange membranes. Under the drive of the bioelectric field, Na^+^ and Cl^−^ selectively permeate into the cathode and anode chambers, respectively, effectively suppressing the co-ion effect. Under optimal operating conditions (external resistance 1000 Ω, initial NaCl concentration 2.0 g/L), the system achieved a cumulative desalination rate of 85.1% after 12 h of operation, with a salt adsorption capacity of 162.1 mg/g, an average desalination rate of 13.51 mg/(g·h), and an energy consumption of only 0.58 kWh/m^3^. This demonstrates that bioelectric energy can effectively provide targeted power to drive capacitive adsorption and desalination. Under initial NaCl concentrations of 1.0 g/L and 3.0 g/L, the highest desalination rates reached 78% and 68%, respectively. The maximum instantaneous desalination rate occurred within 0.5–1.0 h (64 mg/h under 2.0 g/L conditions), exhibiting a three-stage kinetic characteristic of “fast-slow-equilibrium”. The energy consumption in this study was only 0.51 kWh/m^3^, further demonstrating the high energy efficiency of bioelectrically coupled MCDI in low-salinity treatment areas. Therefore, this B-MCDI can serve as a theoretically feasible proof-of-concept technology for desalination of brackish water that meets the requirements of self-driven, low-energy consumption, and has promising applications in decentralized water supply systems in areas with limited energy supply or no available electricity.

## 1. Introduction

With the increasing severity of water shortages due to freshwater scarcity and seawater intrusion, especially in coastal areas and estuaries, the present issue cannot be ignored [1]. According to the current general groundwater quality standards, groundwater with a TDS content of 1 g/L to 3 g/L is called brackish water, which is a transitional type between freshwater and saline water and cannot be directly used as a source of water for domestic and industrial and agricultural production [2]. How to effectively and energy-efficiently desalinate this type of low-to-medium salinity water is an important issue currently facing the water treatment field [3]. Traditional desalination technologies such as reverse osmosis and electrodialysis remain energy-intensive, limiting their economic feasibility in brackish water treatment [4].

In addition to iterative optimization of the microbial desalination battery’s own configuration, coupling the microbial power generation unit with capacitor deionization technology to construct an external desalination system has also become another important technical route [5]. For example, Liang P. [6] used MFC and CDI to treat 60 mg/L low-concentration brine as a classic study, and obtained a desalination rate of 35.6 mg/(L·h), but the charge efficiency was only 21.8%, and the energy utilization efficiency was not ideal [7]. Subsequent studies connected two MFCs in parallel to power MCDI, which increased the electroadsorption capacity to 264.8 μmol/g, but the desalination rate was still only 3.7 mg/h [8]. In order to further enhance the system’s treatment efficiency, in 2012, Zhuang L. et al. further developed a microbial capacitor desalination battery, which directly integrated the capacitor electrode into the desalination system. In the treatment of shale gas produced wastewater, the stack achieved a TDS removal rate of 65% and a COD removal rate of more than 85%, and the desalination rate was 18 times higher than that of the traditional MDC [9]. However, it is worth noting that the above CDI-MFC and MCDC coupling strategies all treat the capacitor deionization unit as an independent external component [10], which is driven by MFC power supply. In essence, it is still an external connection mode of MFC power generation and CDI power consumption [11], rather than the direct embedding of the cathode and capacitor adsorbed at the material and circuit level [12]. There are many intermediate loss links in the energy transmission path [13]. Current research lacks a design that organically couples bio-electro-driven and capacitive adsorption from a circuit perspective [14], namely, establishing a large potential difference to effectively drive ion migration and ensuring a low co-ion concentration under high adsorption rate [15], so as to simultaneously improve desalination rate and energy efficiency ratio and reduce energy consumption [16].

Currently, the main technology for desalination using microbial electrochemical systems is to achieve low-energy desalination of water [17]. However, traditional desalination devices operate by using concentration gradients generated by ion movement, which limits their desalination rate and speed. Combining biological desalination with membrane capacitor deionization is a promising future development direction [18]. To further explore low-cost methods for treating brackish water, this paper attempts to develop a novel microbial anode-membrane capacitor deionization coupling device. The bioelectricity generated by microbial oxidation of organic matter directly powers the electrodes of the MCDI, and electrostatically adsorbs different ions from the brackish water onto the electrode surface to achieve ion separation. It is hoped that organic matter degradation and desalination under low-energy voltage is achieved simultaneously [19]. In summary, based on the need to develop a low-cost, sustainable fresh water desalination technology suitable for treating low-to-medium salinity brackish water, this paper proposes a microbial anode-membrane capacitor deionization coupling system and conducts preliminary construction, mechanism research, and performance studies. This can provide theoretical, performance parameter, and engineering application references for future related technology development [20]. Referring to the terms “Microbial Anode” and “Bioanode” in the literature, which refer to the use of active microbial communities as catalysts as anodes and microbial anodes, and the simultaneous function of membrane capacitive deionization (MCDI) electrodes in conjunction with cathodes 0 [21,22], the microbial anode-membrane capacitive deionization coupling system can be simplified to Bioanode-MCDI, hereinafter referred to as B-MCDI [23].

The B-MCDI architecture constructed in this study differs fundamentally from the three existing technologies. Traditional microbial desalination batteries (MDCs) rely on concentration gradients to drive ion migration, resulting in limited desalination efficiency; microbial fuel cell-capacitor deionization coupling systems (MFC-CDIs) use capacitor deionization as an external load powered by the microbial fuel cell, leading to energy losses; and microbial capacitor desalination batteries (MCDCs), although placing the capacitor electrode in the desalination chamber, do not introduce membrane materials to suppress the co-ion effect. In contrast, B-MCDI directly couples the bioanode and the membrane capacitor deionization cathode at the circuit level, effectively suppressing the co-ion effect using a cation exchange membrane, thereby significantly improving charge utilization efficiency [24,25,26,27,28,29]. The ion mechanism and application of the system were obtained through experiments, which stabilized at a constant resistance value with optimal desalination effect, providing a theoretical basis and experimental reference for subsequent improvements to the system [30].

## 2. Materials and Methods

### 2.1. Experimental Apparatus and Structure Construction

#### 2.1.1. Construction of the Reactor Device Structure

The structure of the microbial anode-capacitor deionization coupling system in this experiment is an extension of the classic three-chamber microbial desalination battery [31]. It consists of three cubic reactor modules, whose basic configuration is mainly based on the cubic MFC reactor proposed by Liu in 2004 [32], and is improved and reconstructed based on the microbial desalination battery reactor proposed by Wang Yi in 2023 [33].

The reactor was made of transparent plexiglass to allow direct observation of the experimental process, while providing the necessary heat, pressure, and corrosion resistance. The three cubic chambers are the same size, each 80 mm long, 80 mm high and 40 mm wide, with a cylindrical cavity of 40 mm diameter in the middle of each chamber. O-rings are embedded in the two sides of the cavity of each chamber to form a plane [34]. The two chambers are tightly attached to prevent leakage of electrode liquid, while increasing the pressure to strengthen the fixation between the chambers [35]. Each chamber has an upper water inlet on one side and a lower water inlet on the other side. In this experiment, the upper part is the water inlet and the lower part is the water outlet. During installation, the water inlets of the three chambers are placed on the same side and the water outlets are placed on the same side [36]. Each chamber has two electrode holes with a diameter of 6 mm at the top of its cubic cavity [37,38,39]. In this experiment, the two electrode holes of the leftmost and rightmost cathode and anode of the entire reactor are connected by shark clips and connected in series with a simple variable resistor box to form an external circuit. The voltage is regulated by adjusting the control resistor and the corresponding current [40].

The reactor consists of three chamber modules, from left to right, corresponding to the left desalination chamber, right desalination chamber, and anode chamber. Their effective volumes are approximately 50.27 mL each, based on the cylinder volume formula [41]. The total volume of the two desalination chambers is 100.54 mL. Both the cathode and anode chambers are covered with solid plexiglass covers. Two identical transparent, sealed plexiglass covers, 80 mm long, 80 mm high, and 10 mm wide, are placed on either side of each chamber to reinforce the reactor and prevent solution overflow [42]. The three chambers are secured together by four 180 mm long M8 double-ended bolts and nuts, and are uniformly tightened to close [43,44].

The cathode electrode is a 50 mm × 50 mm activated carbon sheet [45]. The cathode chamber, located on the left side of the reactor, is constructed by the cathode electrode in close contact with the cation exchange membrane (CEM) [46]. Between these close contacts exists a physical space for containing the micro-cathode liquid, namely the cathode chamber [47]. To minimize the ohmic resistance of the reactor, the chamber widths of the cathode and cation exchange membrane are minimized to reduce the distance between the cathode and anode [48].

The anode chamber contains anolyte, and a 50 mm × 50 mm felt pad for microbial adhesion is placed between the anode chamber and the anode cover plate [49]. A 50 mm × 50 mm anion exchange membrane (AEM) separates the anode chamber containing the anolyte from the desalination chamber, while a 50 mm × 50 mm cation exchange membrane (CEM) separates the activated carbon cathode from the desalination chamber [50].

The cation exchange membranes (CEMs) and anion exchange membranes (AEMs) used in this study were both commercial products. Their identification and manufacturers are as follows: The CEM model was CMI-7000S (Membranes International IncGlen Rock, NJ, USA), a strongly acidic cation exchange membrane with sulfonic acid functional groups, a membrane thickness of 0.45 ± 0.025 mm, an exchange capacity of 1.6 ± 0.1 meq/g, and a resistivity of <30 Ω·cm^2^ in 0.5 mol/L NaCl. The AEM model was AMI-7001S (Membranes International Inc., USA), a strongly basic anion exchange membrane with quaternary ammonium functional groups, a membrane thickness of 0.45 ± 0.025 mm, an exchange capacity of 1.3 ± 0.1 meq/g, and a resistivity of <40 Ω·cm^2^ in 0.5 mol/L NaCl. Both membranes were pretreated by soaking in 0.5 mol/L NaCl solution for 24 h before use.

**Figure 1:**
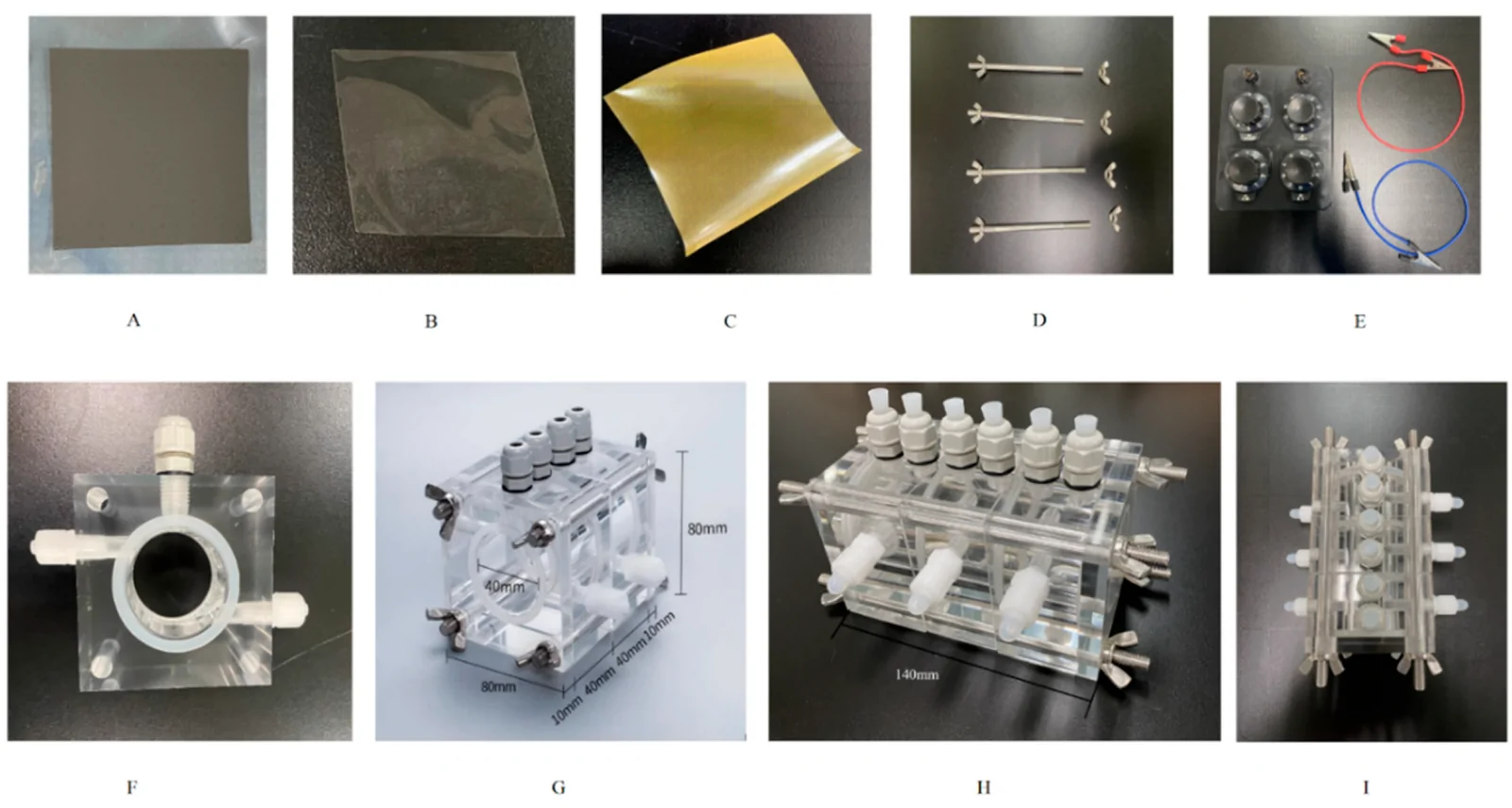
Physical diagrams of the components of the reactor unit. The cathode electrode is shown in (**A**). The cation exchange membrane (CEM) is shown in (**B**), The anion exchange membrane (AEM) is shown in (**C**), The double-headed fixing bolt and nut is shown in (**D**). The simplified variable resistance box and shark clip is shown in (**E**) [ 51]. The side view of the single chamber is shown in (**F**). The two chambers of the overall desalination chamber and the side cover plates and their dimensions are shown in (**G**). The final three-chamber reactor device is assembled, and its front view is shown in (**H**). The top view is shown in (**I**) [52].

The components and dimensions of the reactor device are shown in Figure 1 below.

#### 2.1.2. Preparation and Pretreatment of Microbial Anode Materials

As a receptor that directly receives extracellular electrons, the anode material directly affects the adsorption amount and electron transfer efficiency of microorganisms on the anode, thereby ultimately affecting the electricity production performance of the entire device and system [53]. In the reactor, the anode reaction chamber (hereinafter referred to as the anode chamber) device itself is constructed of custom-made Plexiglas. The selected material for microbial anode preparation is high specific surface area carbon felt [54].

Carbon materials are the most suitable electrode materials to use due to their good electrical conductivity, chemical stability and biocompatibility [55]. In this study, carbon felt was used as the electrode material for microbial attachment, and bacterial suspension was configured as the anolyte in the anode chamber, which serves as the ion adsorption and conduction medium [56]. Carbon fiber felt (HP series, specific surface area >1000 m^2^/g) is spun from carbon fiber filaments with a diameter of 10~25 μm. Due to the three-dimensional network structure formed by weaving the carbon fibers, the carbon felt has many voids and a large specific surface area, which can provide attachment sites for anode electricity-producing microorganisms to the greatest extent and promote high-density organisms. The formation of the membrane allows electrogenic microorganisms to better complete extracellular electron transfer [57]. The internal pore structure of the high specific surface area carbon felt can also accommodate more electrogenic microorganisms, effectively improving the adhesion between microorganisms and electrodes and resisting the erosion effect of hydraulic shear [58]. In addition, the porous structure can also effectively reduce the unit current density on the electrode to reduce polarization and effectively reduce the ohmic internal resistance of the system [59]. Since the carbon felt will have residual oil contaminants and metal elements during the processing process, which will affect the attachment of microorganisms and the extracellular electron transfer process, interfering with the experimental results, especially when operating at low current density, the impact will be great, and pretreatment is required before use [60]. The cut carbon felts were soaked in 2 mol·L^−1^ hydrochloric acid solution for more than 12 h, and then rinsed with deionized water. The carbon felt was then soaked in deionized water and acetone for 12 h respectively. After the last soaking, the carbon felt was subjected to high temperature treatment after deionization and rinse [61]. Heated in a muffle furnace at 350 °C for 30 min, cooled to room temperature and then placed the pretreated carbon felt in a desiccator. Cut to 50 mm × 50 mm, close to the empty chamber and completely cover the circular contact area with a closed diameter of 40 mm [62].

The anolyte design scheme targets electroactive bacteria and electroactive microorganisms to provide a stable electricity-producing environment and minimize the impact on desalination. The nutrient buffer solution used in the experimental anode, referred to as the anolyte, consists of sodium acetate trihydrate (CH_3_COONa·3H_2_O, 1.0 g·L^−1^), 0.15 g·L^−1^ NH_4_Cl, phosphate buffer (PBS, 50 mM, pH 7.0) and 0.01 g·L^−1^ CaCl_2_, 0.01 g·L^−1^ MgCl_2_ trace metal element solution is artificially prepared and composed [63]. Sodium acetate (CH_3_COONa·3H_2_O, 1.0 g/L) serves as a carbon source to provide acetate ions, ensuring a sufficient electron donor. 0.15 g·L^−1^ ammonium chloride provides approximately 39 mg·L-1N in the form of ammonium ions NH_4_^+^, providing necessary nitrogen elements for microorganisms to synthesize proteins, nucleic acids, etc., as a nitrogen source. Be within the appropriate range of microbial nutrition to avoid excessive osmotic pressure burden or additional ion interference [64]. PBS buffer system can maintain pH stability and provide phosphorus. A pH lower than 6.0 will severely inhibit the activity of most electrogenic bacteria. PBS will preferentially neutralize most of the H^+^ to maintain pH stability, while Cl^−^ exists as a counter ion in the anode chamber solution [65]. The metal element solution also directly participates in the electron transfer and energy metabolism of the respiratory chain of microorganisms, maintaining charge balance. All chemical reagents were of analytical grade, and the solutions were prepared with ultrapure water, autoclaved at 121 °C for 20 min, and then stored under anaerobic conditions for later use [66].

#### 2.1.3. Preparation and Pretreatment of Membrane Capacitor Deionization Cathode

The cathode is the main area in the system that accumulates negative charges and absorbs cations. Therefore, the selection and pretreatment of the cathode material are crucial to the desalination effect and stable operation of the system. In this paper, in addition to the function of collecting electrons in the traditional MFC, the cathode also serves as a membrane capacitive deionization (MCDI) electrode, which efficiently captures ions by relying on the double-layer adsorption principle under the action of an electric field. Therefore, the preparation of the cathode must ensure that it has high conductivity, large specific surface area, and good electrochemical and mechanical stability in order to meet the requirements of the CDI process for the electrode [67]. Based on the basic working mechanism of EDI, the selection of the cathode material system is considered from the perspective of the cathode material system. EDI is a desalination technology based on electroadsorption. Its essence is to form a double-layer structure with a high specific surface area by using porous carbon electrode materials (such as activated carbon) under the action of an external electric field, and to achieve the purpose of desalination and concentration of solution through the reversible electrostatic adsorption and desorption behavior of ions [68]. In this system, the cathode and cation exchange membrane (CEM) are placed close together, forming the cathode chamber, located on the left side of the reactor. During system operation, electrons generated by the electrogenic microorganisms in the anode chamber oxidizing organic matter are transferred to the cathode via an external circuit. The negative potential on the cathode surface attracts cations (Na^+^) in the desalination chamber through the cation exchange membrane and is adsorbed on the double layer of the cathode electrode [69]. Anions (Cl^−^) are adsorbed on one side of the anode chamber or enter the anode chamber via the AEM, where they complete the ion migration and separation process. Therefore, the cathode is essentially a composite electrode with both electron receiving and ion adsorption functions, and its performance directly affects the desalination capacity, rate, and energy efficiency of the system [70]. This study uses activated carbon-based composite materials as the cathode electrode. Compared with carbonized materials, activated carbon has a porous structure, high specific surface area, and good chemical stability, resulting in better adsorption performance. The material has a variety of pore structures, including micropores (<2 nm), mesopores (2–50 nm), and macropores (>50 nm). Among these pore types, micropores provide the main adsorption sites, while mesopores facilitate efficient ion transport, and finally, macropores serve as ion buffer regions. The combined effect of these three types can effectively improve the ion adsorption rate and capacity [71].

In this experiment, activated carbon powder was used as the active material for the cathode electrode, carbon black was used to enhance the conductivity of the entire electrode, and polyvinylidene fluoride (PVDF) was used as a binder to ensure the combination of the active material and the current collector, as well as the stability of the electrode structure. The mass ratio of activated carbon: carbon black: PVDF was 8:1:1 (*w*/*w*), which was determined by comprehensively considering the adsorption capacity, conductivity, and mechanical strength of the electrode [72].

**Figure 2:**
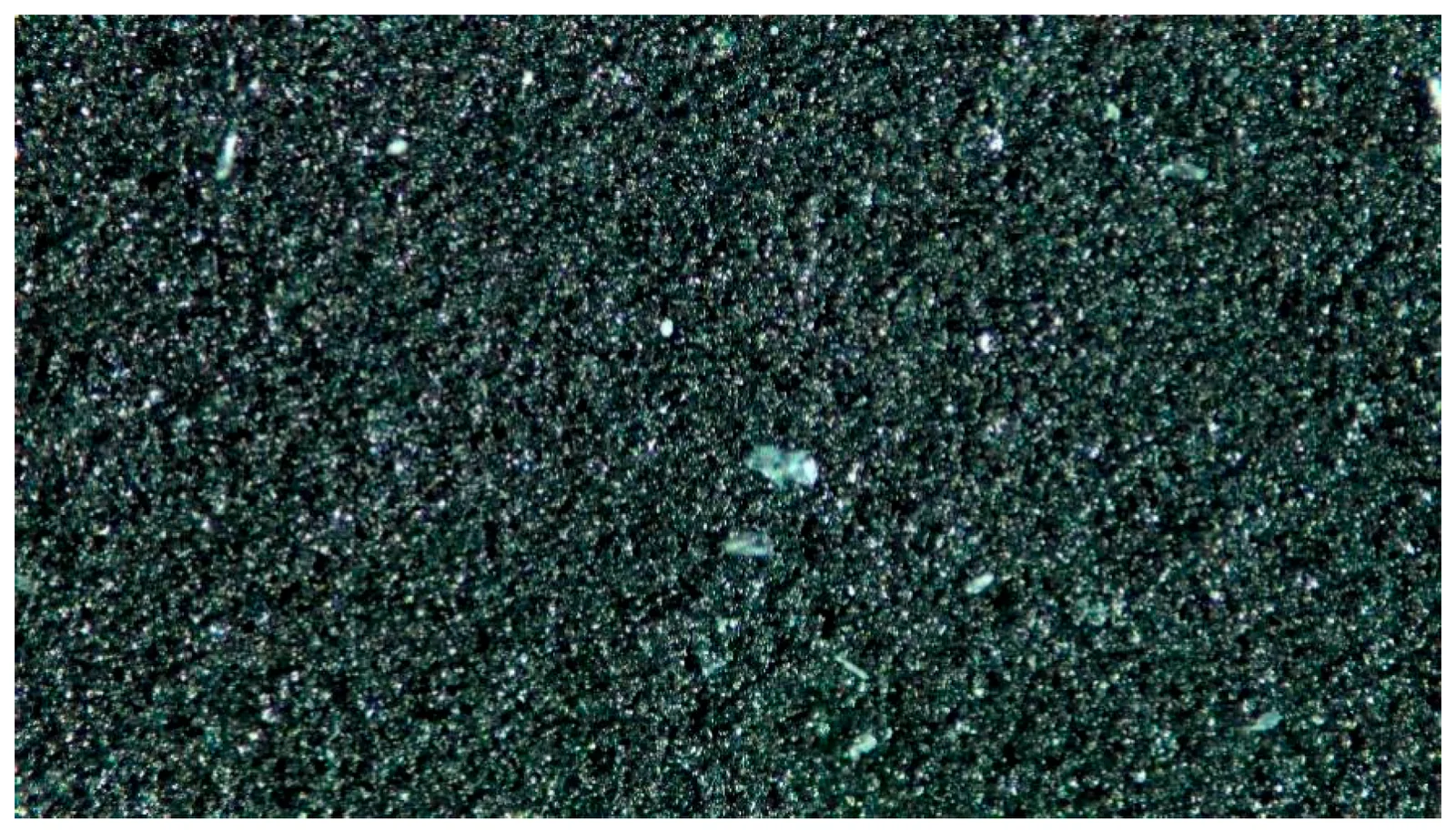
SEM image of the cathode activated carbon electrode surface.

Pre-treatment of the cathode is also required. The freshly prepared electrode may contain impurities or incompletely activated pores, which affect its initial adsorption capacity and subsequent operating performance. Therefore, before assembling it into the reactor, it was electrochemically activated. The electrode was placed in a 0.5 M Na_2_SO_4_ solution. A three-electrode system was used, with the working electrode as the cathode, a Pt sheet as the electrode, and Ag/AgCl as the reference electrode. The cyclic voltammetry (CV) scan conditions were −0.8 to 0 V, with a scan rate of 10 mV/s and 10 cycles [73]. This process is beneficial for electrode surface activation, increasing the effective electrochemical surface area and removing some oxygen-containing functional groups on the surface, thereby enhancing the DLCC and ion adsorption reversibility of the electrode. After activation, the electrode was rinsed multiple times with deionized water to remove the remaining electrolyte and dried in an inert atmosphere for later use [74]. The activated carbon sheet was cut into 50 mm × 50 mm pieces, attached to the cavity and completely covering the 40 mm diameter circular contact surface. The image was acquired under a microscope, and its microstructure is shown in Figure 2. The activated carbon particles are irregularly shaped blocks with a rough surface and a large number of pores. Under 1000× magnification, the pore structure is clearly open, the channels are well connected, the pore size is in the range of submicron to several micrometers, and there are obvious gap channels between the particles.

In the microbial anode-membrane capacitive deionization coupling system, the cathode plays two main roles: first, as the BES terminal electron acceptor, it obtains electrons from the anode microorganisms and ensures the continuous flow of current in the entire external circuit, which is the basis for realizing power generation and energy output [75]; second, as the adsorption electrode of the EDI device, under the action of an external electric field, a strong double layer is generated on its surface, which selectively adsorbs cations in the desalination chamber to remove salt from the water. This process is the same as the traditional membrane capacitive deionization process. The difference is that the driving potential of this device is not provided by the external voltage, but by the biological activity in the anode chamber, that is, the biological energy is directly converted into electrical energy, and this is used to drive the desalination process. In principle, a low-energy consumption or even self-driven desalination method is realized [76].

The functionality of the cathode is realized based on capacitive deionization. Therefore, its functionality should be described from the aspects of electrode specific capacitance, ion adsorption capacity, adsorption kinetics, charge efficiency and cycle stability. The double-layer behavior and cycle life of the electrode were investigated by constant current charge–discharge test; the removal rate and adsorption amount of the target ion by the cathode under different initial salt concentrations and applied voltages were investigated by batch adsorption test, which can establish a quantitative relationship between operating conditions and desalination performance; in addition, the contact resistance at the interface between the cathode and the cation exchange membrane is also one of the key parameters affecting the overall energy efficiency of the system, and it needs to be analyzed by electrochemical impedance spectroscopy [77].

In summary, the cathode is the core part of this coupling system for bioelectrochemical energy conversion and capacitive deionization; the purpose of the preparation and pretreatment of the cathode material is to obtain a porous anode with a large specific surface area, good conductivity and high electrochemical stability, so as to ensure that this coupling system can continuously and effectively use the bioelectricity generated by microorganisms as the driving force for desalination and continuously treat medium and low concentration saline wastewater. Further research is needed on its stable desalination capacity and mechanism under actual conditions, long-term stability and coupling process with microbial anodes [78].

### 2.2. Experimental Methods

To systematically, comprehensively, and accurately evaluate the overall treatment efficiency of the B-MCDI coupling system, this study mainly uses the following chemical detection methods to characterize the influent and effluent water quality and related indicators of different chambers of the reactor [79].

This paper considers the reactor operation from the start-up of newly replenished anolyte to the effluent discharge, with the voltage value dropping to 20% of its highest point, as a batch cycle. During this period, the cycle length of each experimental group will vary according to the bacterial growth. All water samples are collected at the beginning (t_0) and end (t_e) of each batch cycle and analyzed immediately or stored in the dark at 4 °C, with the determination completed within 24 h [80].

(1) Calculation method for capacitive deionization performance

The calculation method for the capacitive deionization performance index is as follows. Based on the above measurement data, the following key performance parameters were calculated to comprehensively evaluate the desalination efficiency and economy of the system.

1. Desalination Efficiency:

Characterized by the degree to which the system removes salt, it is the most direct indicator of desalination performance. It can also be called the cumulative desalination rate. In this paper, the desalination rate is denoted as DE. Its calculation formula is as follows.

(1) $$\mathrm{DE}(\%)=\frac{C_{0}-C_{t}}{C_{0}}\times 100\%$$

*C_0_* and *C_t_* are the salt concentrations (in NaCl, mg/L or mmol/L) in the desalination chamber at the start time t_0 and end time t_e, respectively.

The instantaneous desalination rate (IDR) is the mass of salt actually removed from the desalination chamber per unit time, typically expressed in mg/h.

(2) $$\mathrm{IDR}(t)=-\frac{dm_{\mathrm{NaCl}}(t)}{dt}=-V_{d}\cdot \frac{dC(t)}{dt}$$

*V_d_*: Effective volume of the solution in the desalting chamber (L). In this study, the total volume of the two desalting chambers was 100.54 mL, and *V_d_* was typically taken as 0.1005 L.

*C(t)*: Mass concentration of NaCl in the desalting chamber at time *t* (mg/L). *(dC(t))*/*dt*: The derivative of concentration with respect to time, characterizing the instantaneous rate of concentration change.

2. Salt Adsorption Capacity

This reflects the amount of salt adsorbed per unit mass of adsorbent, i.e., the cathode active material, within one cycle, and is used to measure the efficiency of the electrode material.

(3) $$\mathrm{SAC}(\mathrm{mg}/g)=\frac{(C_{0}-C_{t})\times V_{d}}{m_{\mathrm{cathode}}}$$

where *V_d_* is the volume of the desalination chamber solution (L), and mcathode is the dry weight of the cathode active material (g).

3. Average Desalination Rate

This comprehensively reflects the speed of the desalination process and is a key parameter for evaluating the throughput.

(4) $$\mathrm{MAR}(\mathrm{mg}/g\cdot h)=\frac{\mathrm{SAC}}{\Delta t}$$

Δ*t* is the operating time of one adsorption cycle, in hours (h).

4. Energy Consumption

Assessing the energy consumed by the system to produce a unit volume of fresh water is the core of evaluating its techno-economic efficiency.

(5) $$E({\mathrm{kWh}/m}^{3})=\frac{\int_{0}^{t}U(t)I(t)dt}{V_{d}\times \mathrm{DE}}$$

*U(t)* and *I(t)* are the voltage (V) and current (A) recorded in real time, and the integral of their product is the total electrical energy (J) consumed in that cycle. Dividing by the product of the desalination chamber volume and the desalination rate (i.e., the amount of freshwater produced, m^3^), and converting the units (1 kWh = 3.6 × 10^6^ J), the energy consumption is obtained.

(2) Electrochemical Performance Calculation Methods and Electrochemical Impedance Spectroscopy (EIS)

1. Charge Efficiency

This measures the efficiency of the amount of charge used for desalination. Assessing the actual efficiency of the total charge input to the system used for salt adsorption is a primary indicator of energy utilization. The desalination charge efficiency Λ (%) is calculated as follows.

(6) $$\Lambda (\%)=\frac{F\times (C_{0}-C_{t})\times V_{d}\times M}{\int_{0}^{t}Idt}\times 100\%$$

where *F* is the Faraday constant (96,485 C/mol), M is the molar mass of NaCl (58.44 g/mol), and $\int_{0}^{t}Idt$ is the total charge passing through the system in one operating cycle (coulombs, C), obtained by integrating the current–time curve recorded by the data acquisition system. *C_0_* and *C_t_* are the salt concentrations (mol/L) at the initial time and time t in the desalination chamber, respectively, and *V_d_* is the volume of the desalination chamber (L).

2. Electrochemical Impedance Spectroscopy (EIS)

Electrochemical impedance spectroscopy (EIS) is an electrochemical testing method that, under steady-state conditions, uses a small-amplitude sinusoidal potential or current as a perturbation signal to induce an approximately linear response in the system under test, thereby obtaining the spectral characteristics. This allows for the study of charge transfer and mass diffusion in the electrode system. Due to its minimal interference with the test system, simple mathematical processing, and rich information acquisition, it is one of the most widely used testing methods in the field of electrochemistry. It can be used to analyze the reaction mechanism of electrochemical systems and calculate system kinetic parameters, such as electrode double-layer capacitance, charge transfer resistance, diffusion mass transfer resistance, and ohmic resistance.

In this study, EIS analysis was performed using a dual-electrode method under open-circuit voltage (OCV) in a two-electrode mode. The bioanode served as both the counter and reference electrode, while the MCDI cathode served as the working electrode. The frequency range was 105 Hz to 10^−2^ Hz, with a sinusoidal perturbation amplitude of 5 mV. To ensure data reliability, measurements were repeated three times at each time point, and the average value was used for equivalent circuit fitting. The goodness of fit χ^2^ was less than 1 × 10^−3^. The intercept of the high-frequency curve on the real axis from the obtained Nyquist plot was defined as the total ohmic internal resistance of the system. High frequencies reflect fast processes, such as solution resistance and contact resistance. Low frequencies reflect slow processes, such as diffusion and membrane mass transfer. The cathode served as the working electrode, and the anode served as both the counter and reference electrode.t

## 3. Results and Discussion

### 3.1. Culture Medium Preparation and Strain Domestication

The performance of bioelectrochemical systems mainly depends on the composition of electroactive microorganisms with high efficiency of extracellular electron transfer at the anode interface. Therefore, the screening, activation, expansion and directional domestication of functional microorganisms are the key foundation for constructing high-efficiency microbial anodes. This paper takes the standard model strain of Shewanella oneidensis MR-1 (ATCC 700550) from Lake Oneida as the research object, hereinafter referred to as MR-1. The aim is to construct a stable and reproducible anode bioelectrochemical system [81].

#### 3.1.1. Microbial Activation and Culture

Shewanella oneidensis MR-1 is one of the most widely used bioelectrochemical bacterial models [82]. The experiment used the Shewanella oneidensis MR-1 model strain as the anodic microorganism. Shewanella oneidensis MR-1 is a wild-type strain isolated from the natural environment [83,84].

Strain activation is the first step in reviving freeze-dried strains and restoring their metabolic functions. The MR-1 strain used in this experiment was a commercially available freeze-dried powder. Following aseptic techniques, the freeze-dried powder was activated in a biosafety cabinet. A certain amount of the freeze-dried powder was inoculated into autoclaved nutrient broth. The nutrient broth (NB) consisted of 10.0 g/L peptone, 3.0 g/L beef extract, 5.0 g/L NaCl, and 1.0 g/L glucose, with the pH adjusted to 7.5. Peptone and beef extract provide abundant N, C, vitamins, and growth factors. NaCl maintains an isotonic environment with the microbial cytoplasm. Glucose is a readily available primary C source that can rapidly initiate bacterial metabolism [85]. Commercially available nutrient broth medium was used and placed in anaerobically at 30 °C for 24 h to restore its metabolic activity. The culture after inoculation was placed in a 30 °C constant temperature incubator. The culture was shaken at 150 rpm for 24 h. This aerobic environment is conducive to the rapid reproduction of bacteria. Turbidity of the culture medium indicated successful bacterial activation [86]. The bacteria were cultured in a biosafety cabinet of model NU-437-400E for 24-72 h.

**Figure 3:**
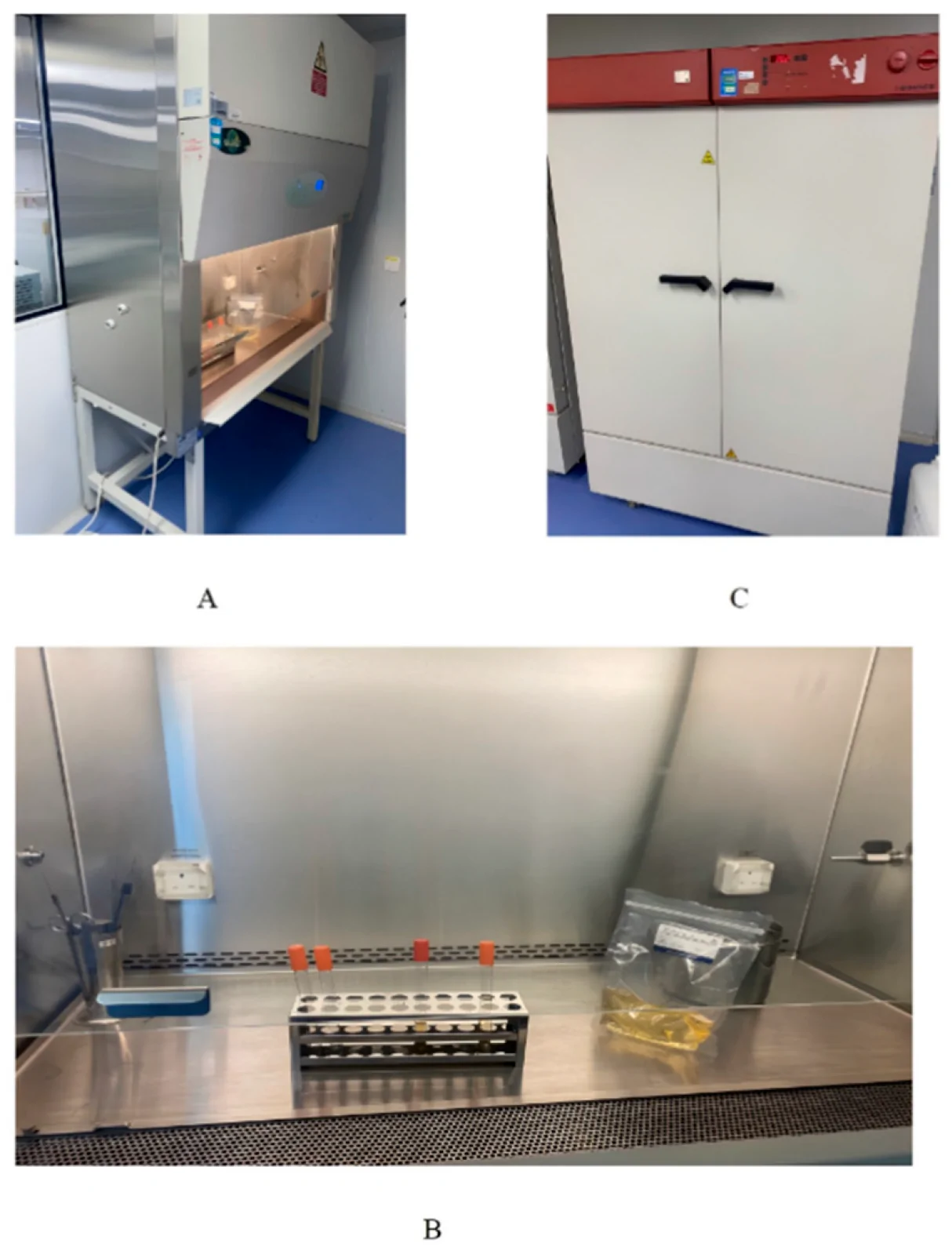
Strain activation and culture. Activation operation test bench (**A**), activation aseptic operation bench (**B**) biosafety cabinet (**C**).

Activation operation test bench, activation aseptic operation bench to biosafety cabinet are shown in Figure 3.

#### 3.1.2. Strain Passage and Culture Medium Selection

In order to obtain a large number of uniformly active bacterial cells for anodic inoculation and to examine their adaptability to different nutrient conditions, it is necessary to subculture the first generation of activated bacterial solution and screen the culture medium. The logarithmic late-stage bacterial solution of the above activated bacterial solution was streaked with an inoculation loop onto four commonly used solid culture medium plates containing nutrient agar (NA), trypsin-soybean agar (TSA), brain heart infusion agar (BHI) and alkaline peptone agar (APW). All plates were placed in a 30 °C incubator and incubated statically for 48 h [87].

**Figure 4:**
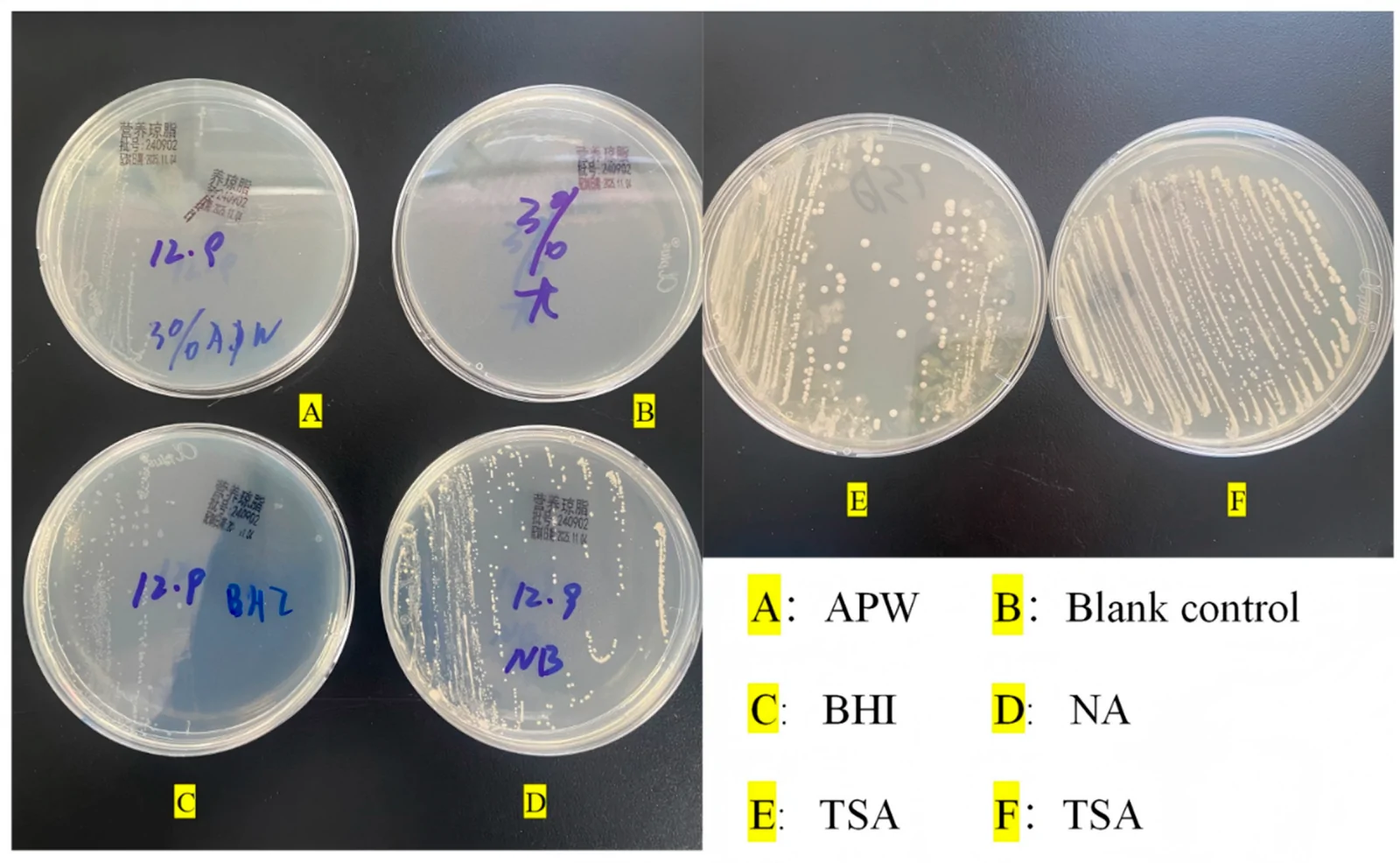
Subculture in different culture media.

The results showed that the strain grew most vigorously on nutrient agar (NA) and trypsin-soybean agar (TSA) plates. However, growth was relatively slow on brain heart infusion agar and alkaline peptone agar, and the colonies formed were small and sparse. This may be due to the different complex nutrients and osmotic pressures of the different culture media. NA and TSA contain a more balanced nutrient composition that is suitable for the rapid growth of MR-1 [88]. The cultivation effects of different nutrient component media are presented in Figure 4.

Based on this result, good single colonies from NA and TSA plates were subjected to second-generation purification culture. Samples were picked from typical single colonies using a sterile inoculation loop and inoculated onto freshly prepared NA and TSA slant media. After incubation at 30 °C for 48 h to form a thick bacterial moss layer, a portion of the moss was picked and spread into a storage reagent tube containing 25% sterile glycerol. After mixing, the sample was stored at −80 °C for later use as a standard strain for subsequent research [89].

**Figure 5:**
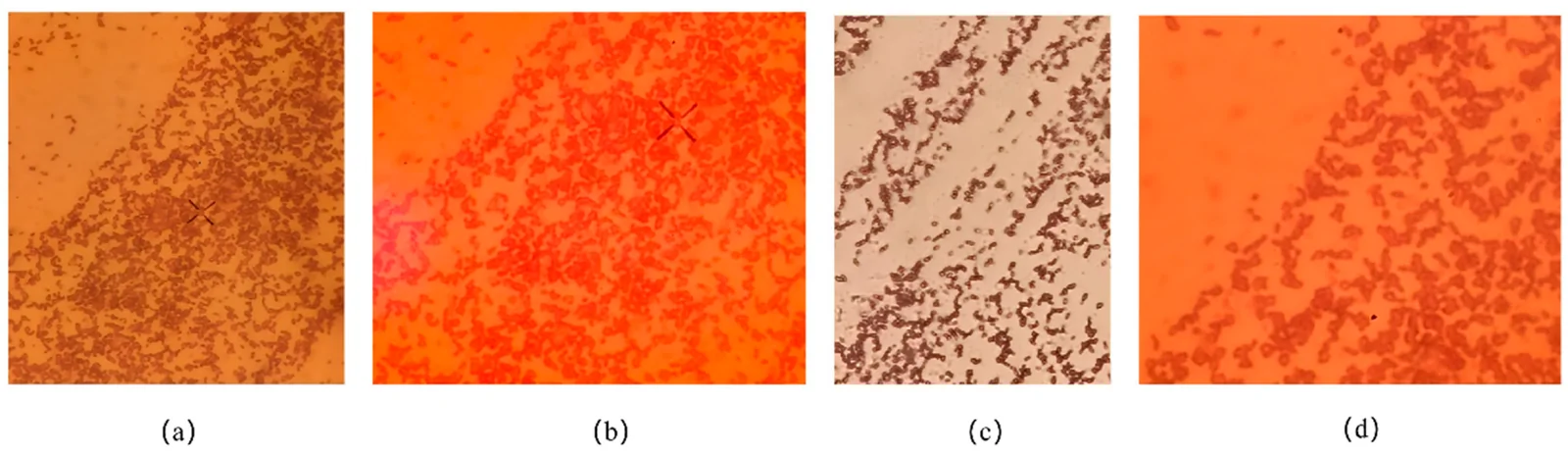
Optical microscopy imaging of MR-1 colonies at different magnifications. (**a**) 100×, overall morphology under low magnification; (**b**) 200×, colony distribution; (**c**) 400×, Gram staining characteristics of bacterial cells (negative, red); (**d**) 1000×, details of short rod-shaped morphology of bacterial cells.

To prepare the anodic inoculum, the bacterial moss from NA and TSA slant media was eluted with sterile physiological saline, centrifuged to collect the bacterial cells, and resuspended in sterile phosphate buffer to prepare a concentrated bacterial suspension with an optical density (OD600) of approximately 1.0. At the same time, typical single colonies on the culture medium were picked using a sterile inoculation loop. After Gram staining, the colony characteristics were gradually magnified under an optical microscope until they could be clearly observed. Gram-negative bacteria appear red. The colonies are dense, round, rod-shaped, raised, and translucent, with a salmon-like color. Figure 5 below shows a clear image of the observed bacterial community.

In this study, Shewanella oneidensis MR-1 showed good growth on both nutrient agar and trypsin-soybean agar, forming characteristic colonies. The colony abundance on both media was roughly equivalent, indicating that these two media could meet the nutritional requirements for bacterial growth [90].

To ensure the reproducibility and consistency of subsequent microbial anode preparation work and considering the routine culture habits of standard model strains in the direction of microbial electrochemistry, this paper determined that trypsin soybean agar (TSA) medium was used as the standard passage and preservation solid medium for MR-1 strain. Firstly, TSA medium has a clear composition and is more nutrient-rich and balanced, which is conducive to the strain maintaining a stable physiological state and electroactivity potential; secondly, compared with the relatively simple NA medium, the multiple nitrogen sources and growth factors provided by TSA may be more conducive to the strain’s tolerance to the potential stress of the anolyte environment during subsequent acclimatization and operation [91].

A single colony with typical morphology was selected from TSA plates for second-generation purification culture to prepare glycerol-preserved bacterial strains, and a homogenized concentrated bacterial suspension for subsequent anodic inoculation was prepared from these strains [92].

#### 3.1.3. Microbial Acclimatization and Anode Configuration

Directly inoculating the enriched bacterial culture onto the anode does not guarantee the immediate formation of an effective electrogenic biofilm. The acclimation process aims to guide and select microbial populations capable of attaching to the electrode surface and efficiently transferring extracellular electrons, adapting them to the target working environment, namely, simulating the chemical composition and electrochemical microenvironment of the anolyte [33].

The acclimation processes described in this paper were all completed in a self-made single-chamber microbial battery acclimation reactor. The reactor anode was a pretreated carbon felt (50 mm × 50 mm). The culture medium used for acclimatization simulated the anolyte in the actual system, which included sodium acetate trihydrate (1.0 g/L) as the sole carbon source and electron donor, ammonium chloride (0.15 g/L) as the nitrogen source, phosphate buffer (50 mM, with phosphate buffer (pH 7.0) to supply phosphorus and maintain pH stability), and trace amounts of calcium, magnesium, iron and other metal ion solutions. Due to the relatively simple composition of the culture medium, a certain nutrient selection pressure was applied to the bacteria to promote the dominant growth of strains that could effectively oxidize sodium acetate and transfer electrons to the solid electrode [93]. The concentrated bacterial suspension of Shewanella oneidensis MR-1 prepared based on TSA culture medium was injected into the acclimatization reactor containing pretreated carbon felt and simulated anolyte at an inoculation rate of 10% (*v*/*v*) to start the directional acclimatization process of the bioanolyte. The external circuit of the reactor was connected through a 1000 A fixed resistor of Ω is connected to the air cathode of the platinum–carbon catalyst to form a closed circuit. The entire system was operated in a 30 °C constant temperature chamber to maintain anaerobic conditions by continuously introducing high-purity nitrogen gas into the anolyte to remove dissolved oxygen, and the anode was placed in the anaerobic chamber for the experiment.

**Figure 6:**
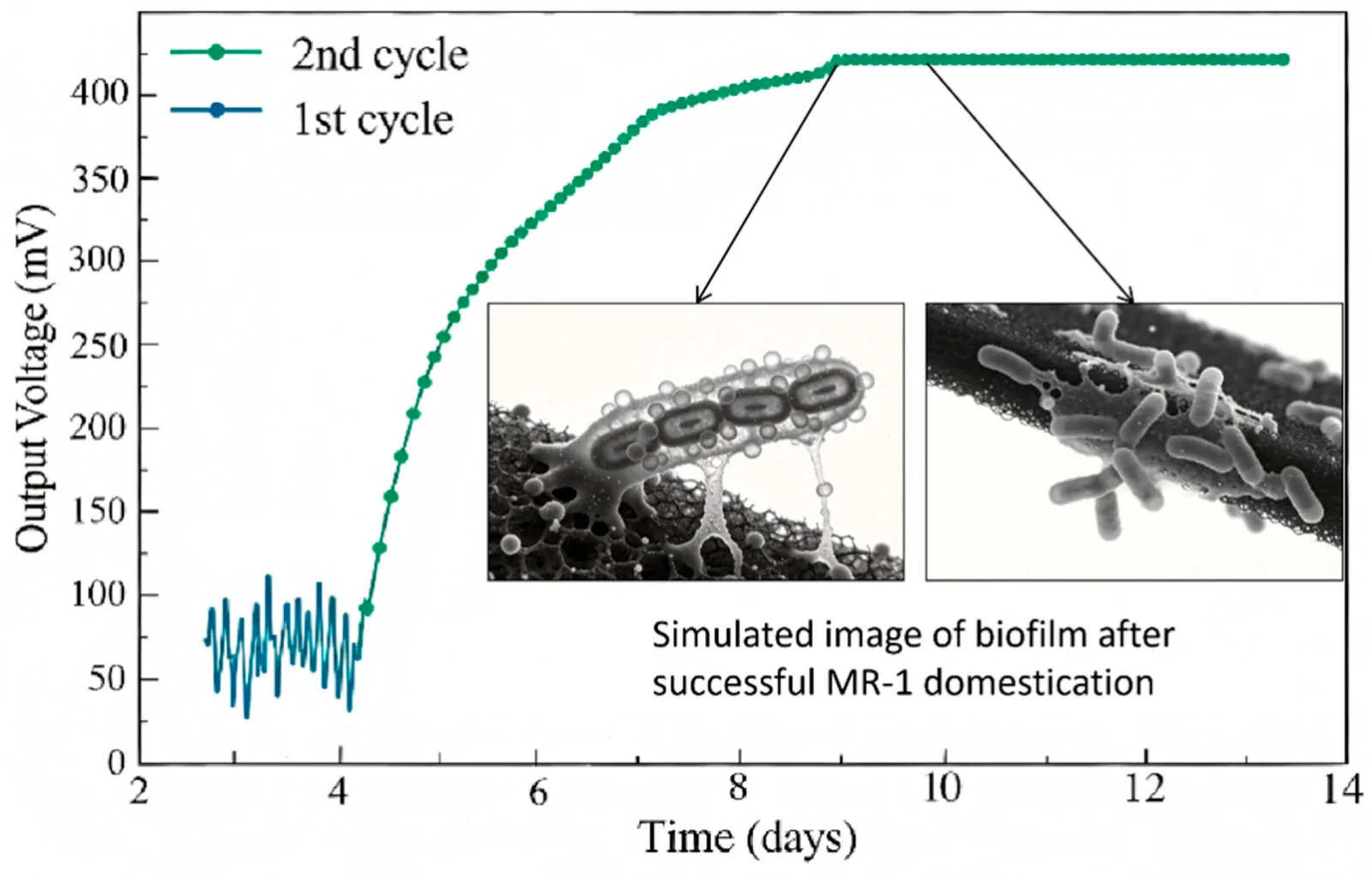
Cyclical changes in strain domestication stability.

The voltage change in the anode chamber is one of the important indicators of biofilm growth and electrochemical reaction. Its change process is shown in Figure 6. It can be divided into the following stages in the entire culture process. The first stage is the initial stage (1–3 d). During this period, the electromotive force generated is very small and in an unstable state, generally less than 200 mV. This is because the bacteria gradually adapt to the anaerobic conditions and gradually adhere to the anode surface. The second stage is the growth stage (3–8 d). The voltage shows a rapid and continuous increase, indicating that the number of electroactive microorganisms attached to the anode has increased, the biofilm has gradually matured, and has an increasingly strong extracellular electron transfer capacity. Finally, the system reaches the stable period (about the 8th day), and the output voltage can reach the expected target, that is, it is stable and greater than 400 mV [94].

The hallmark of successful acclimation is a stable and relatively high system output voltage. Initially, the voltage is low and fluctuates. As electroactive microorganisms capable of attaching to the carbon felt proliferate and form a biofilm, the system voltage gradually increases. When the output voltage remains consistently above 400 mV, and this stable state can be maintained for at least three complete batch operation cycles, the electrogenic biofilm is considered successfully acclimated and has reached a mature steady state, ready for further experiments.

The stability assessment criteria included not only the magnitude of the voltage but also its consistency. During the stabilization period, the system underwent at least three complete batch operation cycles. Specifically, after 2–4 days, the last batch operation cycle was characterized by the output voltage gradually decreasing to below 20% of its maximum value. This voltage level indicates that the microbial community not only degrades the substrate but also establishes an effective extracellular electron transport pathway, providing a considerable current density to the external circuit. After a media change on day 7 after reaching steady state, the voltage rapidly recovers to a high steady-state level. This repeatable voltage recovery capability demonstrates that the electrogenic biofilm has been successfully domesticated and reached a functionally stable state. At this point, the microbial anode possesses the ability to efficiently and continuously convert the substrate’s chemical energy into electrical energy and output it to the external circuit, making it suitable for the subsequent assembly and operation of the B-MCDI coupling system.

After domestication, the carbon felt anode with the mature biofilm was removed. To directly observe whether microorganisms were attached, a sample was taken from the carbon felt, fixed in glutaraldehyde, dehydrated with gradient ethanol, critical point dried, and then characterized using scanning electron microscopy. SEM results showed that a large number of rod-shaped bacteria were attached to the surface of carbon fiber and its gaps, and the cells were connected together by extracellular polymers to form a dense three-dimensional biofilm. This demonstrated the success of domestication from a morphological perspective. Finally, the carbon felt anode loaded with the successfully domesticated MR-1 biofilm was used as the core biological component and installed in a microbial anode-membrane capacitor deionization device (B-MCDI) for further performance and mechanism studies [95].

### 3.2. Establishment of a Microbial Anode and Membrane Capacitor Deionization Coupling System

The core design concept of this system is to utilize the bioelectric energy generated by the oxidation and degradation of organic matter by electroactive microorganisms in the anode chamber as the driving force, which is directly applied to the membrane capacitor deionization electrode in the cathode chamber. Therefore, even without external power input, it can efficiently and actively remove salt from the desalination chamber.

The entire system not only completes the degradation of organic pollutants and energy recovery, but also simultaneously achieves the desalination of low-to-medium salinity water, demonstrating the characteristics of functional synergy and internal energy circulation.

**Figure 7:**
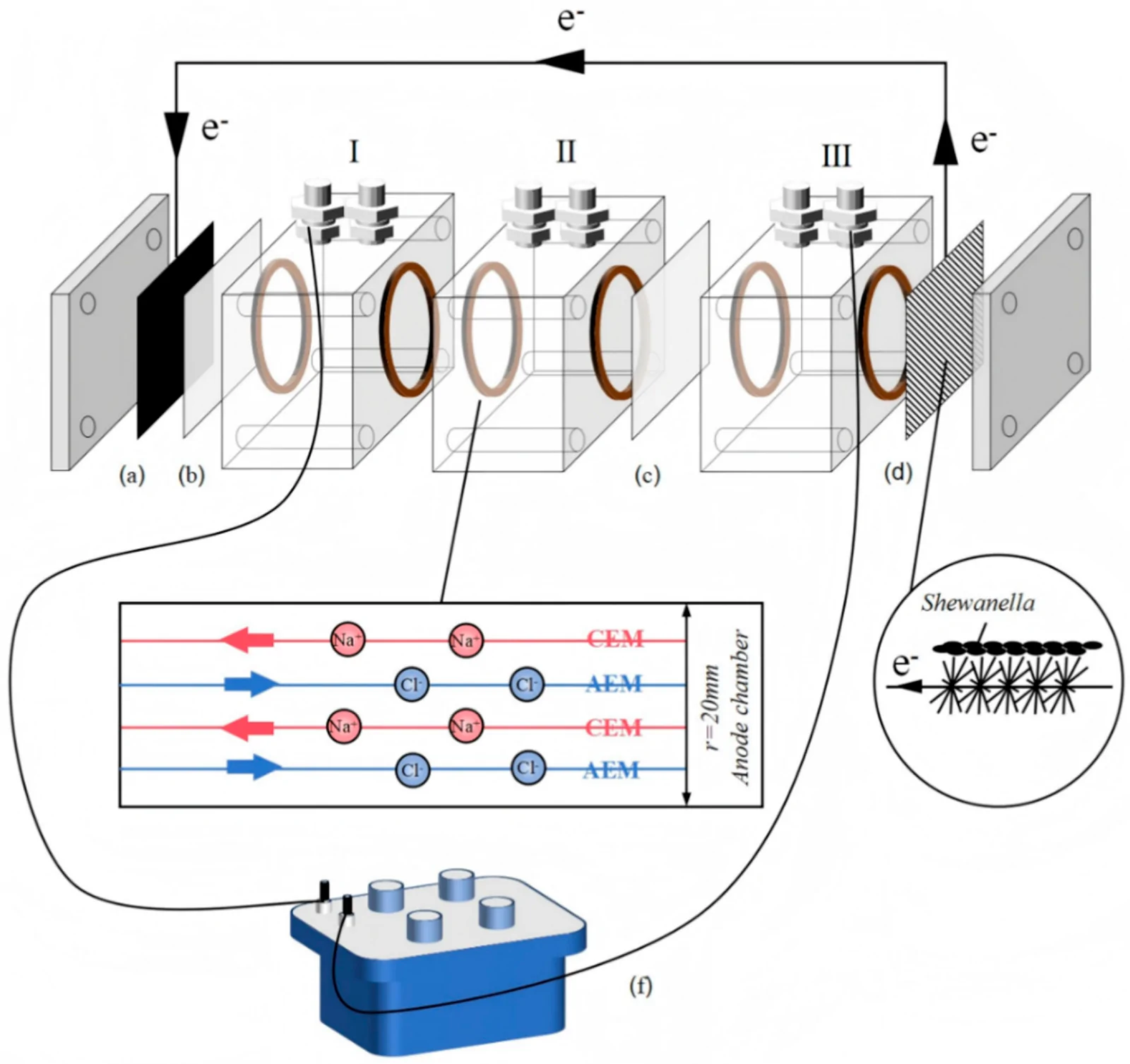
Bioanode-membrane capacitive deionization coupling system. The reactor’s physical configuration is designed and constructed based on a classic three-chamber structure. The overall structure consists of three horizontally arranged chamber units: I (left desalination chamber), II (right desalination chamber), and III (anode chamber). Each chamber is primarily made of transparent plexiglass for easy observation of the internal processes. The leftmost and rightmost plates are the cathode cover and anode cover, respectively. (**a**) Cathode electrode; (**b**) CEM; (**c**) AEM; (**d**) anode felt pad with microorganisms; (**f**) a variable resistor box connected in series with the cathode and anode via shark clip wires, forming the external circuit.

A simplified diagram of the entire coupled system is shown in Figure 7.

**Figure 8:**
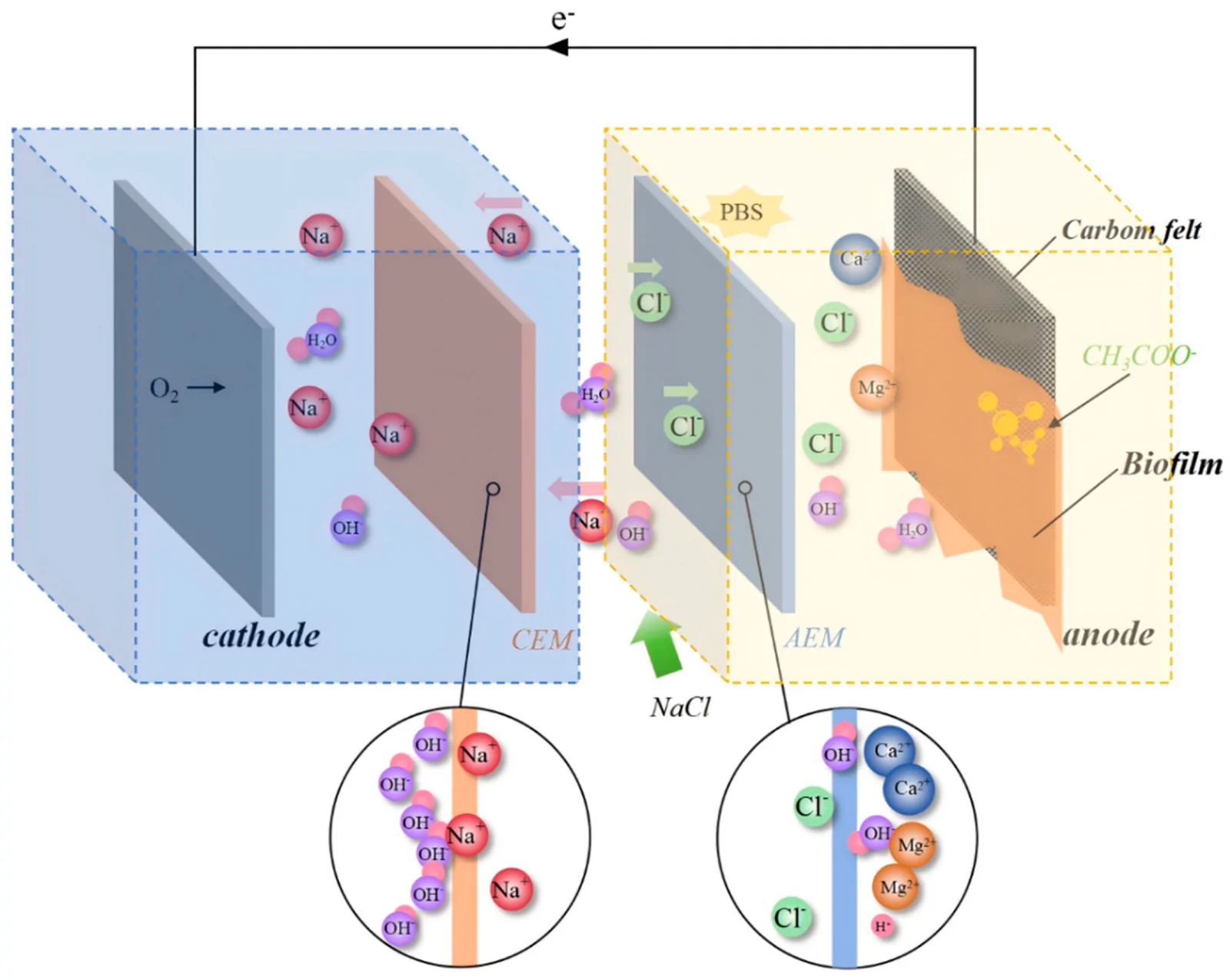
The desalination principle of the Bioanode-Membrane Capacitive Deionization Coupling System.

Capacitive membrane deionization (MCDI) is an evolution of capacitive deionization technology. Its innovation compared to other desalination devices lies in the introduction of an ion exchange membrane between the activated carbon electrode and the solution to be desalinated. In this system, a CEM is used on the cathode side, allowing Na^+^ in the desalination chamber to selectively pass through the CEM when the cathode is polarized (negatively charged) and adsorbed into the electric double layer within the pores of the cathode electrode, forming a physical site for ion storage. Simultaneously, electrons generated by the oxidation of organic matter by electroactive microorganisms in the anode chamber are transferred to the cathode via an external circuit, while the H^+^ accumulated in the anode chamber is balanced by Cl^−^ migrating from the desalination chamber (passing through the anion exchange membrane AEM) to maintain the electroneutrality of the solution. The introduction of the ion exchange membrane effectively suppresses the co-ion effect, i.e., it repels ions with the same charge as the electrode, thereby improving charge utilization efficiency and desalination capacity. During the discharge regeneration stage, by short-circuiting or reversing the electrode polarity, the ions stored in the electric double layer can be rapidly released, achieving electrode regeneration and concentrate discharge. A simplified diagram of the principle is shown below on Figure 8.

### 3.3. System Stability Testing and Reactor Start-Up

Shewanella is a facultative anaerobic bacterium that can perform aerobic respiration under aerobic conditions, while generating electricity under anaerobic conditions through various types of terminal electron acceptors, such as solid electrodes. However, the primary mechanism of electricity generation in the B-MCDI process is extracellular electron transfer, and the reaction mainly occurs along the anaerobic respiration pathway, resulting in high efficiency. When oxygen is present in the anode chamber, the microorganisms, using oxygen as a readily available electron acceptor, will not transfer electrons to the electrodes, leading to low or no voltage and current levels in the system. Therefore, although the bacterial species itself is aerobic/facultative anaerobic, efficient electricity generation requires an anaerobic environment to transfer electrons to the electrodes. During the acclimation and system operation phases of the experiment, the anode chamber should be strictly anaerobic.

After completing the acclimation of the microbial anode and the physical assembly of the reactor, rigorous sealing verification and system startup are performed to ensure the reliability of experimental data and the stability of reactor operation. Physical sealing is a fundamental prerequisite for the reactor’s functionality. Therefore, during the assembly process, silicone rubber gaskets were placed between each ion exchange membrane and the contact surface of the plexiglass cavity to enhance the seal. When tightening the bolts, the principle of alternating diagonal tightening and uniform tightening at each stage was followed to ensure uniform stress on the membrane surface and avoid leakage or membrane damage caused by local stress. After assembly, a pressure test was performed to verify the sealing effect [96].

The test involves injecting a colored aqueous solution containing methylene blue into the anode chamber, allowing it to stand for more than 24 h, and closely observing whether the solution and cover plate in the adjacent desalination chamber and cathode chamber turn blue. Only after confirming that there was no dye leakage could the reactor be deemed physically sealed and ready for biological and electrochemical experiments.

First, the mature carbon felt anode loaded with a stable electrogenic biofilm was installed into the reactor anode chamber. Sterilized anolyte, which has been bubbled with nitrogen for at least 30 min to completely remove dissolved oxygen, is injected into the anode chamber. Simultaneously, a continuous trace amount of nitrogen is maintained to create and maintain a strictly anaerobic working environment for the electrogenic microorganisms. This aims to inhibit the facultative anaerobic Shewanella from using oxygen for respiration, forcing them to use the anode as the terminal electron acceptor, thereby efficiently outputting current [97].

The system output voltage and current are continuously recorded by a data acquisition system. When the system exhibits repeatable voltage output and decay curves within 2–3 batch cycles, and the voltage shows regular periodic changes, it indicates that the B-MCDI coupling system has successfully started up and entered a stable operating state, and subsequent studies on ion migration behavior and electrochemical mechanisms can begin. Furthermore, when it indicates that the process corresponds well with the substrate consumption process within each batch cycle, the reactor startup is considered complete, the system has entered a stable operating state, and subsequent formal experiments under set operating conditions can begin.

### 3.4. Desalination Effect and Performance Analysis of B-MCDI Coupling System

#### 3.4.1. Desalination Effect of the B-MCDI Coupling System

The system’s operating voltage and current are not artificially set, but rather a dynamic balance determined by the output capacity of the bioanode and the internal resistance of the MCDI unit. To maintain the continuity of the system’s microbial anode operating environment, this study initially allowed the biofilm to smoothly transition from a stable resistance state to the coupled system, avoiding performance fluctuations or degradation caused by abrupt changes in electrochemical conditions, and observing the system’s desalination performance. The system was gradually started at a higher resistance of 2000 Ω, and then at a moderate resistance of 1000 Ω, resulting in a higher output voltage and a smaller, more moderate current. This facilitated clear and stable monitoring of the voltage build-up process after circuit closure and safe observation of whether the system could smoothly enter the electrolytic desalination cycle. Based on the acclimatization phase experiments and relevant literature, after the system had stabilized at 1000 Ω for several cycles, gradually adjusting the resistance to lower it did not achieve the same effect on ion migration rate, desalination efficiency, and electrode process stability as at 1000 Ω. The highest and most stable desalination efficiency was achieved when the system was set to a stable value of 1000 Ω within the variable resistance box. The following desalination effect studies were then conducted.

Based on a clear understanding of the basic operating mechanism and electrochemical characteristics of the B-MCDI system, the influence of key operating parameters on the system’s desalination performance was recorded. All experiments were conducted after the system had been stably running for at least three cycles at an external resistance of 1000 Ω to confirm that the system had reached a stable operating state, thus ensuring the reliability of biofilm activity and system status.

The system was continuously run for three complete batch cycles at an external resistance of 1000 Ω and an initial salt concentration of 2.0 g/L, indicating that the system had reached stability before formal performance testing. A complete batch cycle began with replacing the fresh solution in all chambers (anode, desalination, and cathode) and starting the system, and ended when the system output voltage naturally decayed from its peak value to 20% of that peak value. The cycle durations were 8.8 h, 9.1 h, and 8.9 h, respectively, with desalination rates of 80.5%, 81.2%, and 81.3%. The relative standard deviations of all indicators were less than 5%, indicating that the system had reached a stable operating state. After three consecutive cycles, the biofilm of the microbial battery can be considered stable.

The influent salt concentration is one of the core factors affecting the desalination performance of the B-MCDI system. This study selected three representative concentration gradients within the low to medium salinity range to conduct experiments, comprehensively evaluating the system’s applicability to brackish water treatment. The initial NaCl concentrations in the desalination chamber were set at 1.0 g/L, 2.0 g/L, and 3.0 g/L, corresponding to the lower, median, and upper limits of the brackish water range, respectively. With other operating conditions kept constant, the external resistance was 1000 Ω, and the composition of the anolyte was the same as described above. The experiment was formally conducted and measured using the above experimental methods. All experiments were repeated three times. Overall statistics show that the relative standard deviation (RSD) is always less than 5%. The error bars in the following images are not shown, which also shows that the experimental data has good repeatability.

**Figure 9:**
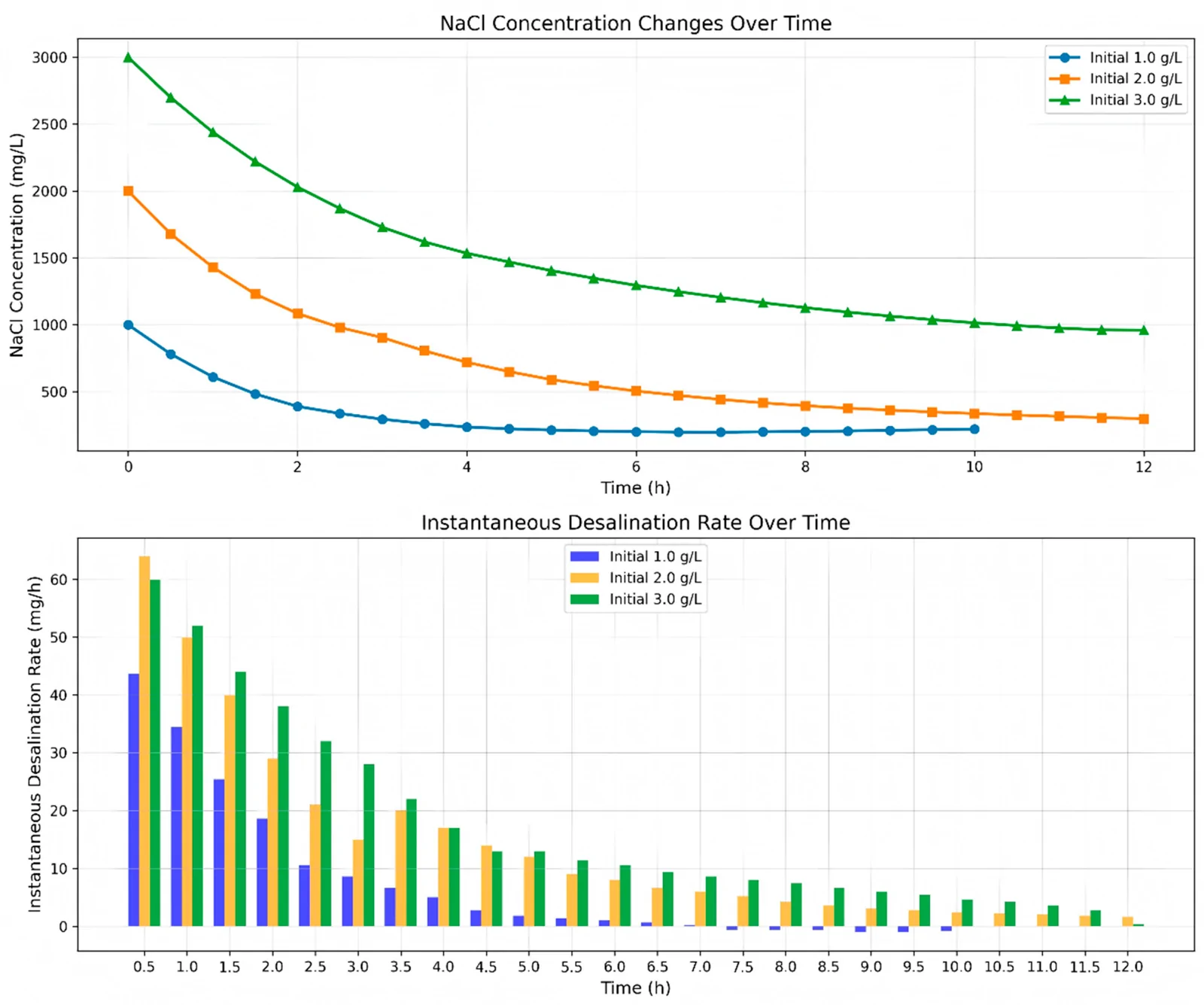
Changes in NaCl concentration and IDR in the desalination chamber under different initial concentrations.

Figure 9 shows the NaCl concentration in the desalination chamber under different influent salt concentrations as a function of operating time. The IDR is obtained based on the central difference (i.e., the boundary is forward or backward). As shown in the figure, for all three concentration conditions, desalination in the desalination chamber is initially rapid and then slows down. In the initial stage of desalination, the driving force comes from the strong electric field exerted on the MCDI electrode when the bioelectricity is just established. Under high voltage, the MCDI cathode rapidly captures Na^+^ almost through forced adsorption, resulting in a sharp decrease in conductivity and a rapid reduction in the C/C_0_ value.

When the influent concentration was 1.0 g/L, the salinity dropped to below 0.198 g/L after approximately 6.5 h of operation, reaching the upper limit of total dissolved solids in the drinking water standards. At an influent concentration of 2.0 g/L, the concentration dropped to 0.298 g/L after approximately 12 h; and at an influent concentration of 3.0 g/L, the concentration dropped to 0.960 g/L after approximately 12 h, indicating a deep desalination process.

Cumulative desalination rates show that at 1.0 g/L, the residual concentration was 220 mg/L, representing a DE of 78.0%; at 2.0 g/L, the residual concentration was 298 mg/L, representing a desalination rate of 85.1%, close to 86%; and at 3.0 g/L, the residual concentration was 960 mg/L, representing a desalination rate of 68.0%. The instantaneous rate peak occurred between 0.5 and 1.0 h, consistent with the characteristics of the current rise phase in a bioelectrochemical system. The peak IDR at 2.0 g/L (64 mg/h) is higher than that at 1.0 g/L (43.6 mg/h), but lower than that at 3.0 g/L (60 mg/h). The non-monotonic relationship between peak IDR and salinity reflects the balance between increased initial conductivity and inhibited microbial activity at high salt concentrations.

From the data and the graph showing the change in IDR, the boundary between Positive IDR (desorption dominated) and Slight Negative IDR (concentration rebound) is essentially that when IDR > 0, the system is still dominated by desalination, i.e., ion migration; when IDR < 0, the NaCl concentration in the desalination chamber increases again, i.e., a concentration rebound occurs. The turning point is the time when IDR first becomes negative.

Starting at a concentration of 1 g/L, the IDR remained positive for 0–7.0 h, dominated by desorption and migration, with a rapid decrease in desalination rate but still effective. Around 7.5 h, the IDR reversed to negative, showing a slight concentration rebound, indicating insufficient driving force and the presence of back diffusion, concentration polarization release, electromigration decay, and adsorption site rebalancing. Specifically, the IDR of 1.0 g/L showed a slight negative value between 7.5–9.5 h, a slight concentration rebound due to ion back diffusion or measurement error, indicating a possible concentration increase. Higher ion concentrations increase the concentration difference between the desalination chamber and the anode and cathode chambers, potentially causing back diffusion. High salinity had a mild inhibitory effect on the metabolic activity of Shewanella; the average current at 3.0 g/L was 412 μA, lower than the 455 μA at 2.0 g/L.

**Figure 10:**
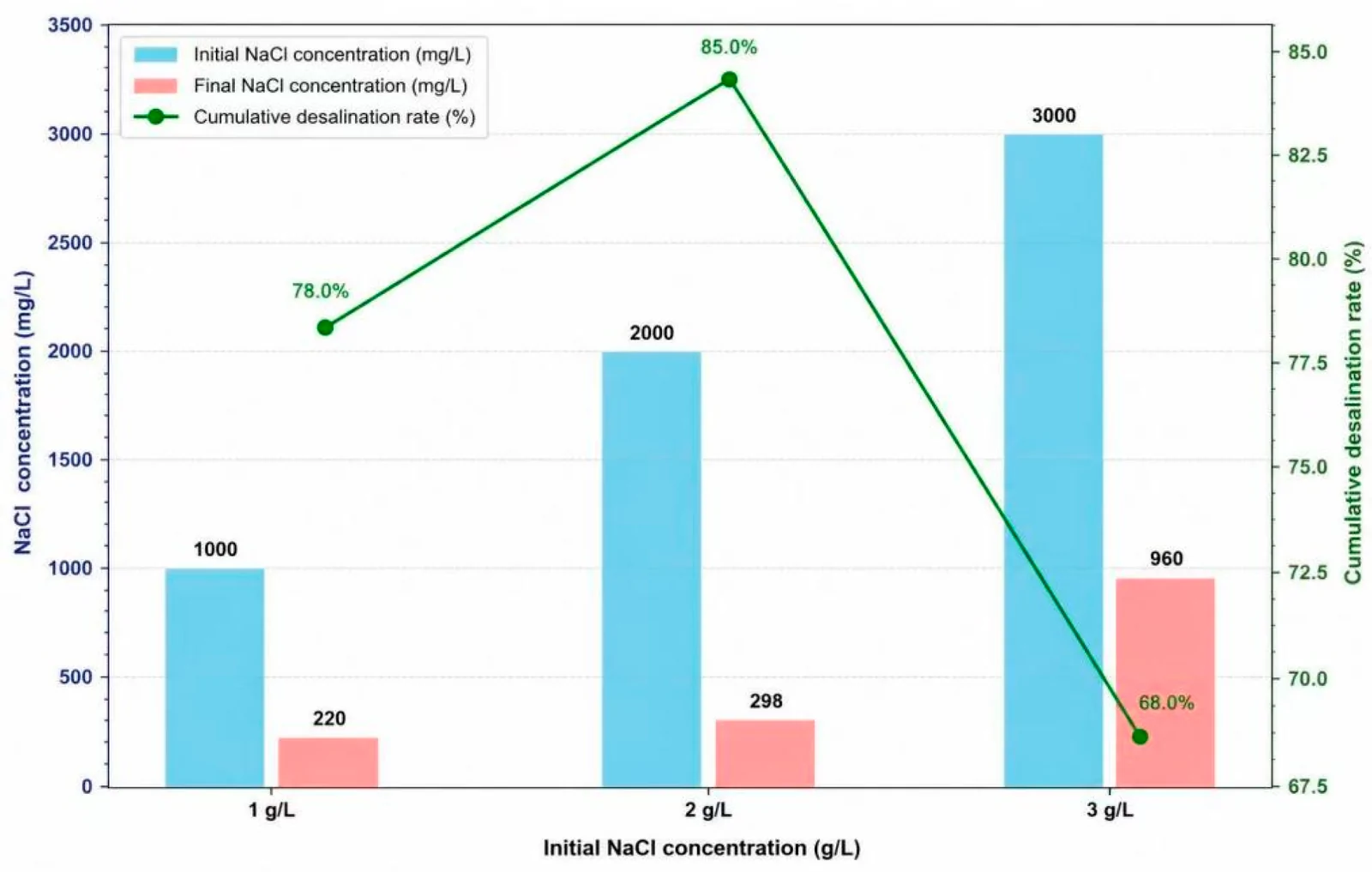
NaCl concentration analysis and cumulative desalination rate (DE).

**Figure 11:**
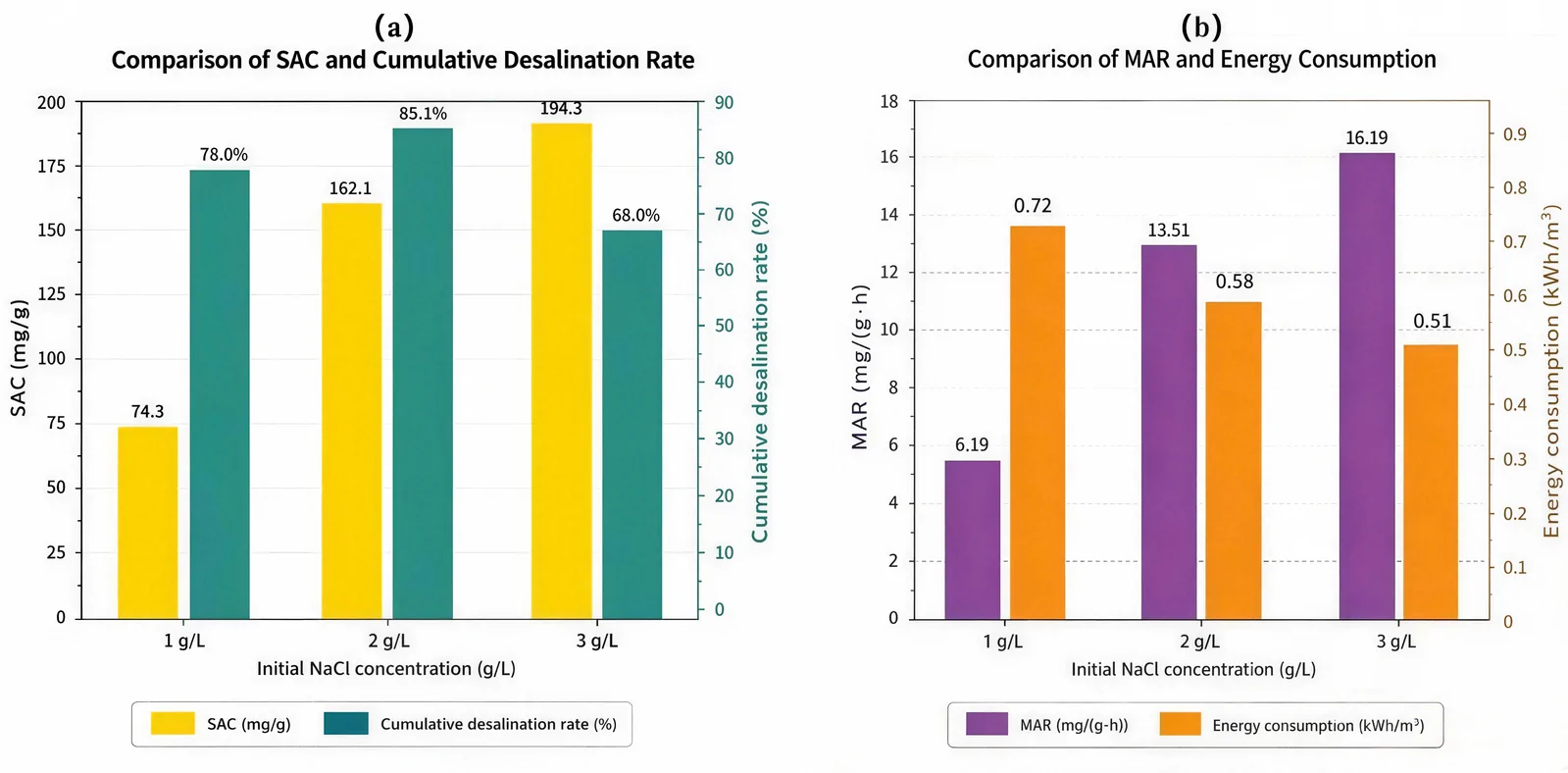
Cathode SAC, cumulative desalination amount, MAR, and energy consumption at different initial NaCl concentrations. (**a**) The figure shows the cathode SAC and the cumulative desalination amount of the system. (**b**) The figure shows the MAR and energy consumption.

At 2 g/L and 3 g/L, the IDR decreased significantly, followed by a slow desalination period. An initial concentration of 2.0 g/L exhibits the highest IDR, but the desalination efficiency decreases slightly afterward at 3.0 g/L. The system reaches near equilibrium. Near equilibrium, reversible adsorption–desorption occurs in the electrode double layer, causing the instantaneous rate to fluctuate near zero. Higher initial concentrations result in higher IDRs and a longer initial forward rate. Most desalination occurs within the first 0–3 h, and after approximately 6–8 h, the driving force decreases, leading to a dynamic equilibrium. The changes in NaCl concentration and instantaneous desalination rate (IDR) in the desalination chamber under different initial concentrations are shown in Figure 10. Other indicators can be found in Table 1 and Figure 11.

**Table 1:** Desalination performance indicators of B-MCDI system under different initial salt concentrations.

Initial Salt Concentration (g/L)	Stable Concentration After 12 h (mg/L)	Cumulative Desalination Rate DE (%)	Desalination Amount Δm (mg)	Salt Adsorption Capacity SAC (mg/g)	Average Desalination Rate MAR (mg/(g·h))	Energy Consumption per Unit Volume E (kWh/m^3^)
1.0	220	78.0	78.0	74.3	6.19	0.62
2.0	298	85.1	170.2	162.1	13.51	0.58
3.0	960	68.0	204.0	194.3	16.19	0.38

SAC increases monotonically with increasing initial salt concentration, from 74.3 mg/g at 1.0 g/L to 194.3 mg/g at 3.0 g/L, an increase of approximately 1.6 times. This trend is consistent with the adsorption isotherm law of porous cathodes; a higher bulk salt concentration provides a greater supply of salt ions, allowing for a higher degree of filling of the double-layer adsorption sites on the surface of the cathode activated carbon material. However, the change in DE is not completely synchronized with SAC. DE reaches 85.1% at 2.0 g/L, significantly higher than 78.0% at 1.0 g/L and 68.0% at 3.0 g/L. This indicates that DE depends not only on the SAC value, i.e., whether the adsorption sites are filled, but also on the total amount of initial salt ions. When the initial concentration is moderate at 2.0 g/L, the match between adsorption capacity and total ion volume is optimal, allowing the system to remove the majority of salt with high output efficiency. However, when the initial concentration further increases to 3.0 g/L, although the salinity concentration (SAC) increases further, the absolute desalination rate as a percentage of the total decreases significantly, reflecting factors such as inhibited biological activity or limited ion migration at high salinity.

The maximum adsorption rate (MAR) is highly positively correlated with the initial concentration. A higher initial salt concentration means that a larger amount of Na^+^ and Cl^−^ can be driven to migrate in the desalination chamber. Under conditions of constant biomass electrolysis and good biofilm condition, more ions can be adsorbed per unit time. However, the MAR does not directly characterize the desalination depth of the system. Although the MAR is highest at 3.0 g/L (16.19 mg/(g·h)), the final desalination efficiency (DE) is only 68.0%, and the effluent salinity is still as high as 960 mg/L, far exceeding the 298 mg/L of the effluent at 2.0 g/L. This indicates that the desalination speed is fast but not clean enough.

Energy consumption (E) decreased with increasing initial salt concentration, from 0.62 kWh/m^3^ at 1.0 g/L to 0.38 kWh/m^3^ at 3.0 g/L. Higher initial salt concentrations enhance solution conductivity, which helps reduce the system’s ohmic resistance, allowing the same bioelectrical work to drive ion migration with lower energy loss, thus reducing the energy cost per unit volume of freshwater produced. Current research on energy consumption of MCDI systems in the 0–2 g/L TDS range indicates that energy consumption is closely related to operating parameters and influent characteristics, with a unit volume energy consumption of 0.89–2.74 kWh/m^3^, and the optimal operating condition being below 1 kWh/m^3^. The energy consumption of 0.58 kWh/m^3^ at 2.0 g/L in this study falls within this optimal range and is significantly lower than the lower end of the range, indicating that this system has certain advantages in terms of energy efficiency.

The cumulative desalination rate reached a maximum of 85.1% at 2.0 g/L, but due to the high initial concentration, the absolute removal amount was also greater. The cathode SAC increased with increasing initial concentration, ranging from 74.3 to 194.3 mg/g, consistent with the electrode adsorption isotherm. The average desalination rate (MAR) was highest at an initial concentration of 3.0 g/L, reaching 16.19 mg/(g·h), but also exhibiting the lowest energy consumption, indicating higher desalination efficiency per unit energy consumption at high salinity, but ultimately insufficient desalination depth. In summary, an initial NaCl concentration of 2.0 g/L and an external resistance of 1000 Ω achieved the optimal balance between desalination rate, SAC, and energy consumption, representing the optimal operating condition for this system. Under this condition, the B-MCDI coupling mechanism achieved optimal energy and mass matching between bioelectricity generation and capacitive adsorption, balancing high desalination depth and excellent energy utilization efficiency, demonstrating its application potential as a low-energy self-driven technology in the field of brackish water desalination.

#### 3.4.2. Desalination Performance and Mechanism Analysis of the B-MCDI Coupling System Compared with Traditional Benchmarks

The performance data reveals the following ion migration mechanism in this system. Taking the 2.0 g/L operating condition as an example, firstly, the NaCl concentration in the desalination chamber exhibits a three-stage decay pattern of “fast-slow-stable,” which closely matches the current–time curve, indicating that the ion migration rate is directly regulated by biocurrent. Secondly, under the 2.0 g/L operating condition, the calculated desalination charge efficiency reaches 93.9%, meaning that over 93% of the charge transferred by the external circuit is used to drive ions to cross the ion exchange membrane and adsorb onto the cathode double layer. This high proportion indirectly demonstrates that the co-ion effect is effectively suppressed; that is, if the co-ion effect is significant, a large amount of charge will be wasted due to the repulsion of like ions and will not be driven to generate charge efficiency. Third, under the condition of 2.0 g/L, the pH of the cathode chamber increased from 7.0 to 8.4, which was positively correlated with the total charge passing through the cathode. This is consistent with the stoichiometric relationship of the water reduction reaction (2H_2_O + 2e^−^ → H_2_ + 2OH^−^), indicating that most of the electrons flowing into the cathode were used for the target electrochemical reaction rather than dissipated through side reactions or leakage paths. Fourth, the Cl^−^ concentration in the anode chamber increased from the initial 105.5 mg/L to 2082.5 mg/L at the end of the cycle, which basically matched the decrease in Cl^−^ in the desalination chamber, indicating the selective permeation of Cl^−^ by the anion exchange membrane. See the Table 2 below.

**Table 2:** Desalination charge efficiency (Λ) of the B-MCDI system under different initial salt concentrations.

Initial Salt Concentration (g/L)	Total Charge Qtotal (C)	Desalination Amount Δm (mg)	Theoretical Charge Qtheo (C)	Charge Efficiency Λ (%)
1.0	10.1	78.0	128.7	78.4
2.0	14.75	170.2	280.7	93.9
3.0	17.8	204.0	336.5	88.5

The concentration Λ (deionization potential) reached its highest value (93.9%) at 2.0 g/L, indicating that the efficiency of bioelectrical energy conversion to desalination work was highest at this salt concentration. Λ was lower at 1.0 g/L (78.4%), suggesting that the total ion content was limited and some charge was not effectively utilized. Λ decreased at 3.0 g/L (88.5%), suggesting that side reactions and co-ion effects were exacerbated at high salinity.

Instantaneous Λ was highest in the early stage (2–5 h) of operation (>95%), and decreased later due to current decay and reduced ion concentration. Cumulative Λ gradually increased and then stabilized (approximately 94%). Data indicate that the co-ion inhibition effect was relatively more significant at 2.0 g/L.

**Figure 12:**
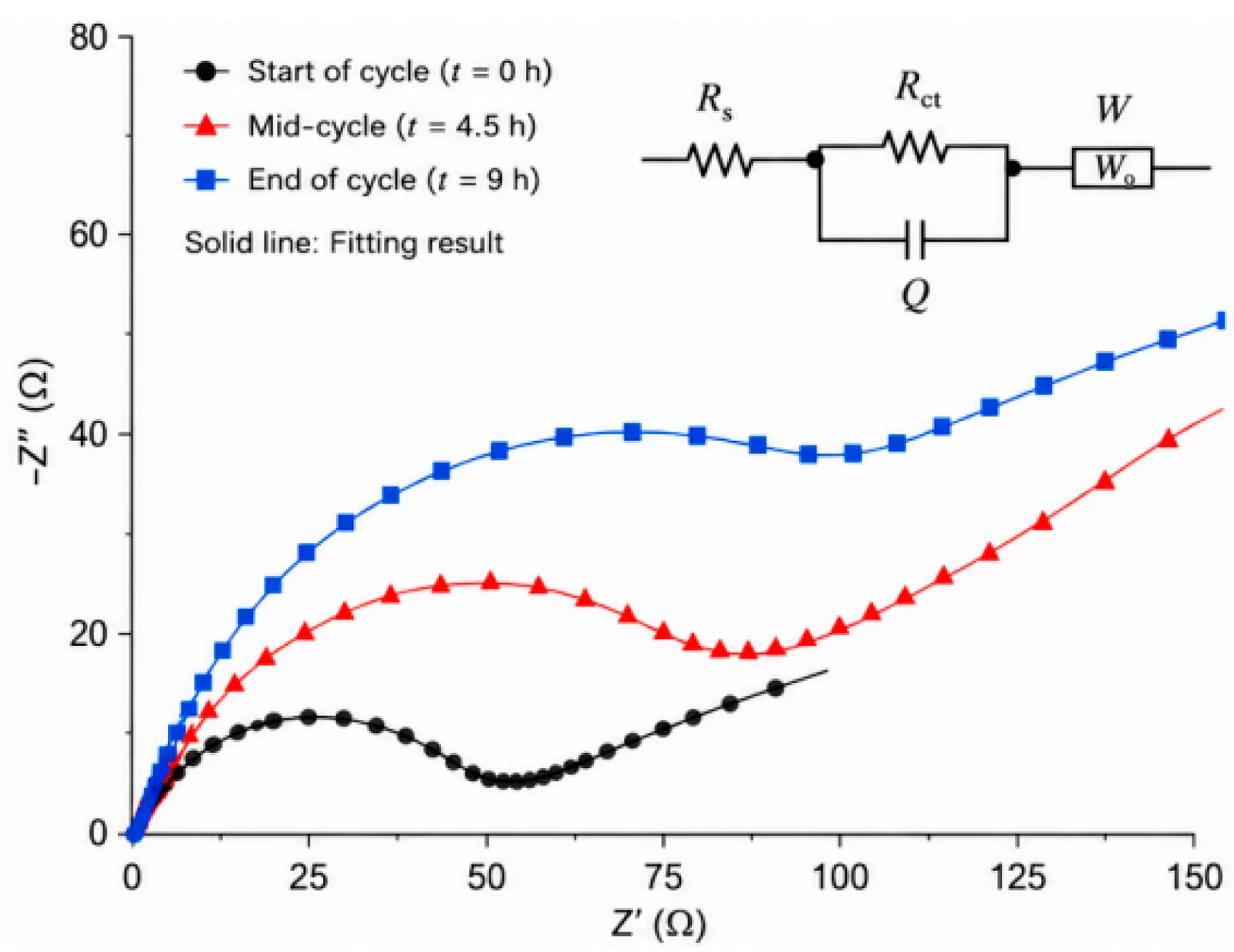
Nyquist plots of the B-MCDI system at different operating stages (external resistance 1000 Ω).

A complete chain of mechanistic explanation can be achieved by combining electrochemical impedance spectroscopy (EIS) analysis, which can quantitatively distinguish the contributions of solution resistance, charge transfer resistance, and diffusion impedance, and is a key tool corresponding to the hypotheses of “co-ion inhibition” and “ion migration pathways.” The evolution of internal resistance of the B-MCDI system during its operating cycle was revealed. As shown in the figure, the Nyquist plot consists of a semi-circular arc in the high-frequency region and a 45° oblique line in the low-frequency region, corresponding to the charge transfer process and the diffusion mass transfer process, respectively. A typical operating cycle under the conditions of an initial salt concentration of 2.0 g/L and an external resistance of 1000 Ω was used as a representative operating condition. An equivalent circuit Rs(RctQ)W was used for fitting, and the goodness of fit χ^2^ was less than 1 × 10^−3^. The smaller the χ^2^ value, the higher the agreement between the fitted curve and the experimental data points. The parameter values obtained for each stage are listed in Table 3. The internal resistance parameters for each stage are also shown in Table 3, Figure 12.

R_s_ represents the ohmic resistance of the solution, including the resistance of the bulk solution, the ion exchange membrane, and the interface between the electrode and the membrane. R_ct_ represents the charge transfer resistance at the anolyte/biofilm interface, reflecting the resistance to electron transport kinetics at the anolyte/biofilm interface. Q is a constant phase angle element (characterizing the non-ideal behavior of the electric double layer capacitance), and W is the Warburg diffusion impedance coefficient (characterizing the resistance to ion diffusion mass transfer). R_ct_ and W increase significantly with operating time, indicating that both charge transfer and diffusion mass transfer are limited in the later stages of desalination, with diffusion impedance dominating.

At the beginning of the cycle (t = 0 h), the total internal resistance was 42.9 Ω, with R_ct_ (22.3 Ω) dominating, reflecting the kinetic resistance to electron transport at the anodic biofilm interface. As operation progressed to the middle of the cycle (t = 4.5 h), R_ct_ increased to 38.7 Ω, an increase of approximately 73%, consistent with the decrease in microbial electrogenic rate due to substrate consumption. Simultaneously, W increased from 12.1 Ω·s^−0.5^ to 28.5 Ω·s^−0.5^, reflecting the increased diffusion mass transfer resistance due to the decreased ion concentration in the desalination chamber. By the end of the cycle (t = 9 h), the total internal resistance reached 115.2 Ω, and W was 45.8 Ω·s^−0^.^5^. Compared to the internal resistance of conventional MDCs (150–250 Ω), the total internal resistance of this system remains relatively low, mainly due to three factors. The high conductivity of the MCDI cathode activated carbon material reduces the electrode resistance, indirectly indicating that the cation exchange membrane effectively suppresses the co-ion effect and reduces interfacial polarization; the compact electrode spacing (<1 mm) significantly reduces the ohmic resistance of the solution.

The total internal resistance of this system (42.9–115.2 Ω) is significantly lower than that of conventional MDC (150–250 Ω), mainly due to the low resistance of the MCDI cathode and the effective suppression of the co-ion effect by the ion exchange membrane. EIS analysis illustrates the advantages of the “bio-electrogenesis-capacitive adsorption” coupling strategy in reducing system internal resistance and improving charge transport efficiency from the perspective of electrochemical impedance spectroscopy.

Further focusing on the initial performance characterization stage of the coupled system as demonstrated in this study, the key desalination performance indicators of this system are compared with those of similar systems in the literature as follows.

Regarding desalination efficiency, in 2024, Koók L et al. conducted a 24 h batch operation comparative experiment, using a three-compartment MDC with acetate-cultured mixed bacteria to achieve a desalination efficiency of approximately 40.3% for 35 g/L NaCl [98]. The desalination rate of this B-MCDI, 85.1%, is significantly higher than the literature value of 40.3% for traditional MDC, an improvement of approximately 2.1 times. Another study used a five-compartment MDC with a LaNi_5_/C cathode catalyst to evaluate its catalytic performance for the oxygen reduction reaction. It achieved a maximum desalination efficiency of 92.29%, but the operating cycle was as long as 72 h. This system achieved a desalination efficiency of 85.1% for 2.0 g/L NaCl within a 9 h cycle, showing a significant advantage in time efficiency [98]. In addition, in 2025, five independent tests were conducted using brine from the Caspian Sea and the Persian Gulf, as well as wastewater from urban wastewater treatment plants, to optimize the performance of MDC by integrating machine learning prediction, thermal imaging analysis, and electrochemical monitoring. A salt removal efficiency of 96% was achieved, but this also requires a long operating cycle and a complex optimization process [99]. In a 2025 study by Liu et al. at Bioresource Technology, three configurations of MDC (PMDC, CMDC, AMDC) all achieved a desalination efficiency of over 97%. However, it should be noted that this study was conducted on high-salinity brine with 35 g/L NaCl, and during long-term operation, polyvalent cations induced a 21.4% loss in desalination efficiency and a 38.3% decrease in power. This also indicates that high desalination efficiency is often accompanied by a long operating cycle and membrane fouling costs [100]. In terms of energy consumption, the study compared the results with the MCDI system in the literature. Cañas Kurz et al. used a pilot-scale MCDI device to investigate the effects of different operating parameters on desalination performance under 0–2 g/L NaCl (TDS) conditions. The energy consumption of the electrode section ranged from 0.89 to 2.74 kWh/m^3^, with the optimal operating condition being less than 1 kWh/m^3^ [101]. The system’s energy consumption per unit volume at an initial salt concentration of 2.0 g/L is 0.58 kWh/m^3^, which is at the low end of this range, indicating that bioelectrically driven capacitive adsorption has certain potential in terms of energy economy.

This difference mainly stems from the fundamental difference in architecture. In MFC-CDI, the CDI acts as an external load, resulting in multiple energy losses through energy conversion and transmission between MFC power generation and CDI adsorption [102]. In contrast, this system directly uses the MCDI cathode as the terminal electron acceptor in the bioelectrochemical system, eliminating intermediate energy loss links. Due to differences in reactor configuration, electrode materials, bacterial strains, and operating conditions, this system serves as a performance reference and is not a strict control. Future research could conduct control experiments under the same geometric dimensions and load conditions to further quantify the incremental value of this coupling strategy.

At the same time, the incremental value of this B-MCDI coupling strategy will be evaluated. Referring to the above and previous literature on similar technologies and structures, the system performance will be compared after the control experiments have stabilized. As shown in the picture below.

**Figure 13:**
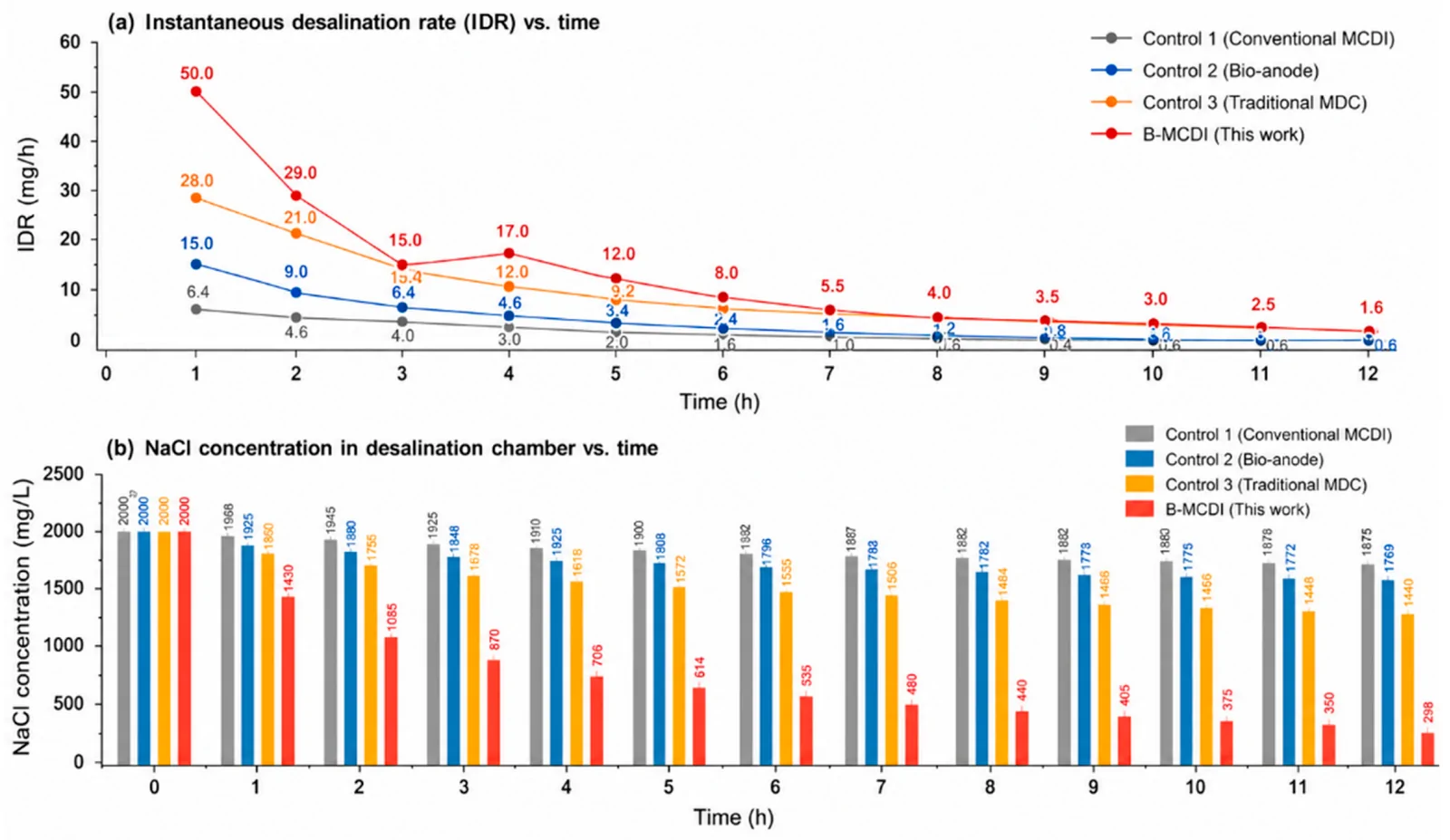
Changes in NaCl concentration and IDR in three control groups and the B-MCDI system.

All three control experiments and the B-MCDI system were run under identical geometric dimensions in a three-chamber reaction structure, with a two-chamber desalination chamber (total 100.54 mL). They were all operated under the same conditions of 1000 Ω external resistance and an initial NaCl concentration of 2.0 g/L, with an observation period of 12 h for each. Each control group and the B-MCDI system underwent three independent replicate experiments. In the supplementary control experiment, three sets of baseline conditions were set: (1) Uncoupled MCDI (i.e., disconnected external circuit and no resistor connected), the desalination rate was only 7.8% within 12 h. It can be seen that the passive diffusion contribution of the ion exchange membrane is minimal when there is no electric field driving, and the desalination effect is not obvious; (2) only single bioanodine operation and ion exchange membrane, without capacitive adsorption function (removal of MCDI capacitor layer), the desalination rate was 18.4%, indicating that bioelectricity generation cannot effectively adsorb to the cut-off ions when there is no capacitive adsorption; (3) traditional three-chamber MDC (replacing the MCDI cathode with ordinary carbon cloth cathode, other configurations unchanged), under the same geometric size and 1000 Ω external resistance, the desalination rate was 43.7% within 12 h, which is significantly different from the 85.1% of this B-MCDI [102]. The above control experiments show that the embedding of the MCDI cathode increases the desalination rate by about 1.95 times. The desalination efficiencies of the control experiments were as follows: uncoupled MCDI 7.8% ± 0.6%, bioanodine only 18.4% ± 1.2%, conventional MDC 43.7% ± 2.1%, and B-MCDI system 85.1% ± 1.8% (n = 3, mean ± SD). One-way ANOVA showed significant differences between B-MCDI and the three control groups (*p* < 0.05), see Figure 13.

Regarding stability, this study conducted 15 consecutive batch-cycle stability tests under conditions of 1000 Ω external resistance and an initial salt concentration of 2.0 g/L. The cycle durations were 8.8 h, 9.1 h, and 8.9 h (the first three cycles), with the duration of the subsequent 12 cycles fluctuating between 8.5 and 9.5 h. The desalination rates in the first three cycles were 80.5%, 81.2%, and 81.3%, respectively, with a relative standard deviation of <5%. At the end of the 15th cycle, the desalination rate remained above 95% of the initial value (77.5%). This stability level is similar to the short-term operating performance of MDC. However, the stability tests in this study only covered a cumulative operating time of approximately 135 h, which is insufficient to assess the long-term scaling and performance degradation of the ion exchange membrane. Ion exchange membranes experience a significant decrease in ionic conductivity during long-term operation. Furthermore, the activated carbon electrode of the MCDI cathode may face a decrease in adsorption capacity and irreversible fouling after multiple adsorption–desorption cycles. Membrane fouling characterization and electrode regeneration cycle testing have not yet been conducted on this system; these will be the focus of future research. At this stage, the stability conclusions of this system should be limited to reproducible performance under short-term batch operation conditions, rather than long-term engineering operation stability.

## 4. Conclusions

This study successfully constructed a bioanode-membrane capacitive deionization (B-MCDI) coupling system, and systematically demonstrated the technical feasibility of using bioelectricity to directly drive capacitive adsorption for brackish water desalination on a small-scale, batch-scale laboratory basis. The main conclusions are as follows.

(1) Construction and Operation Mechanism of B-MCDI Coupled System

Based on a three-chamber reactor configuration (anode chamber, dual desalination chamber, MCDI cathode chamber), Shewanella oneidensis MR-1 from Lake Oneida was used as the model electrogenic bacterium. After directional domestication, a stable electroactive biofilm was formed on the carbon felt anode surface. The bioanode was directly coupled to the activated carbon-based MCDI cathode via an external circuit, realizing a synergistic energy and material pathway of “bioelectricity generation—electric field drive—capacitive adsorption”. During operation, Na^+^ in the desalination chamber was adsorbed by the cathode double layer through the cation exchange membrane, while Cl^−^ passed through the anion exchange membrane into the anode chamber to maintain charge balance. The ion exchange membrane effectively suppressed the co-ion effect, resulting in a significantly higher desalination charge efficiency than traditional microbial desalination batteries.

(2) Desalination performance, optimal operating conditions and low-cost desalination scheme for low-to-medium salinity brackish water

Under the conditions of an external resistance of 1000 Ω and a NaCl concentration of 2.0 g/L, the B-MCDI achieved the highest desalination efficiency. In a typical operating cycle (approximately 9 h), the NaCl solution concentration in the desalination chamber decreased from 2000 mg/L to 298 mg/L, with a cumulative MAR of 85.1%. The IDR reached a maximum of 64 mg/h during the first 0.5–1.0 h and decreased over time, indicating a “rapid rise-stable desalination-approaching limit” variation pattern in the desalination process. The cathode SAC was 162.1 mg/g, the cumulative desalination amount was 170.2 mg, the MAR = 13.51 mg/(g·h), and the energy consumption per unit volume of freshwater was 0.58 kWh/m^3^.

Under different influent salinity levels (1.0, 2.0, 3.0 g/L), the 2.0 g/L salinity condition achieved a balance between high desalination rate, adsorption capacity, and low power consumption, with a cumulative desalination rate of 85.1%, significantly higher than the 78.0% and 68.0% at 1.0 g/L and 3.0 g/L, respectively. As influent salinity increased, the salinity concentration (SAC) increased (74.3 → 162.1 → 194.3 mg/g), but the effluent salinity at 3.0 g/L remained at 960 mg/L, indicating relatively low desalination capacity. With increasing influent salinity, the mean average adsorption rate (MAR) per unit time increased (6.19 → 13.51 → 16.19 mg/(g·h)), but a high MAR does not necessarily indicate a high desalination rate. Energy consumption decreased with increasing salt concentration (0.62 → 0.46 → 0.38). Under the conditions of 2.0 g/L NaCl concentration (kWh/m^3^), the system achieves low energy consumption while ensuring effluent quality meets standards.

An initial NaCl concentration of 2.0 g/L and an external resistance of 1000 Ω represent the optimal operating conditions for this system. Under these conditions, the B-MCDI coupling mechanism achieves the optimal material and energy match between bioelectricity generation and capacitive adsorption, resulting in a high cumulative desalination rate, moderate salt adsorption capacity, and economically feasible unit energy consumption. This demonstrates the feasibility of this technology as a low-energy, self-driven solution for brackish water desalination.

The B-MCDI system requires no external power source, utilizing only the organic matter in the wastewater as an energy source to simultaneously achieve organic matter degradation and desalination. Its unit volume energy consumption (0.38–0.62 kWh/m^3^) is significantly lower than that of traditional reverse osmosis (above 2.2 kWh/m^3^) and electrodialysis technologies, and its energy consumption is far lower than traditional desalination technologies. This allows for expansion to areas with insufficient power supply or high energy costs. For the problem of brackish water (1–3 g/L) pollution caused by saltwater intrusion along the Chinese coast, this system can serve as a new, decentralized, low-cost solution for treatment.

(3) Feasibility limitations and technological prospects

Although B-MCDI has high energy utilization efficiency, its absolute desalination flux is relatively small due to the inherent limitation of low bio-electricity power density. The absolute desalination flux is limited by the bio-electricity power density. The key breakthrough for future engineering is to improve the anode coulombic efficiency, but this can be achieved through nano structuring of electrode materials, synergistic metabolism of mixed microbial communities, and scale-up using electrode stacking or parallel reactor connection. Research should be conducted on coupling with other technologies such as solar energy and microbial electrolysis cells to achieve continuous operation around the clock.

The B-MCDI system established in this paper can also serve as a novel proof-of-concept technology prototype for efficient energy-targeted utilization in a self-driven, low-energy-consumption, high-desalination-rate low-salinity brackish water desalination system based on natural potential gradient changes. This system is currently in the laboratory batch pilot stage. At an initial salt concentration of 3.0 g/L, the effluent salt concentration is still as high as 960 mg/L, which does not yet meet drinking water standards. The prescribed total dissolved solids limit is 1000 mg/L, and actual high-quality drinking water typically requires even lower limits. Even under the optimal operating condition of 2.0 g/L, the effluent concentration of 298 mg/L meets drinking water standards, but the system’s treatment flux (MAR = 13.51 mg/(g·h)) and total treated water volume (<0.1 L per cycle) are extremely limited.

Before the system can be declared stable for engineering deployment, future engineering scale-up will require longer cycling times to address issues such as increasing bioelectricity power density, reactor stacking and continuous flow operation, membrane fouling assessment, and performance degradation after multiple electrode regeneration cycles.

In small communities, industrial parks, and agricultural and livestock areas lacking centralized wastewater treatment facilities, organic wastewater can be directly used as the anode substrate to simultaneously treat domestic sewage and desalinated brackish water. The desalinated water produced by the system can meet various non-potable water requirements, such as irrigation, industrial cooling, and municipal mixed use. This achieves on-site recovery and recycling of water resources and energy. It has broad application potential in water-scarce coastal and inland areas worldwide, and with future engineering scale-up and performance optimization, it will become a technological reserve and application prospect for stepwise water treatment and energy recovery.

## Figures and Tables

**Table 3:** Changes in EIS fitting parameters at different operating stages.

During Operation	Solution Resistance R_s_ (Ω)	Charge Transfer Resistance R_ct_ (Ω)	Diffusion Impedance W (Ω·s^−0.5^)	Total Internal Resistance R_total_ (Ω)
Beginning of the period (t = 0 h)	8.5	22.3	12.1	42.9
Middle of the period (t = 4.5 h)	12.8	38.7	28.5	80.0
End of the period (t = 9 h)	18.2	62.3	45.8	115.2

## Data Availability

The data presented in this study are available from the authors upon request.
